# Supplementary material for: Modality independent or modality specific? Common computations underlie confidence judgements in visual and auditory decisions
Source: PLoS Comput Biol. 2023 Jul 14;19(7):e1011245. doi: 10.1371/journal.pcbi.1011245 (PMC10426961; doi:10.1371/journal.pcbi.1011245)
Supplement: S1 Text — Fig A. Control Experiment: Categorisation Accuracy as a Function of Stimulus Intensity. Table A. Model Fits for Control Data. Table B. Parameter Settings Across Modalities for Control Data. Fig B. Model Comparison for Control Study. (DOCX) [file pcbi.1011245.s001.docx]

**S1 Text: Control Study**

**Fig 3** and **Fig 5** show that for the visual tasks, categorisation accuracy increased approximately linearly with increasing stimulus intensity, as expected. However, in the auditory tasks, the same pattern was not observed. That is, categorisation accuracy was closer to chance at the bottom two intensity levels and then around 75% for the top two intensity levels. To ensure that the difference in first order task performance did not account for the observed modelling results, we repeated the auditory different SDs task in 4 participants. We used different intensity levels (equivalent of -3 dB SPL, 4 dB SPL, 11dB SPL, 17 dB SPL for a 1 kHz pure tone) for the auditory task which were chosen based on the results of the main study and further piloting. Furthermore, we did not correct frequencies for equal loudness prior to adjusting stimulus intensity. This allowed for better comparison with the visual task in which orientation-based adjustments were not made to stimulus contrast. As intended, in the control study categorisation accuracy in the auditory task was approximately linearly increasing with stimulus intensity, as in the visual task (see **Fig A**).


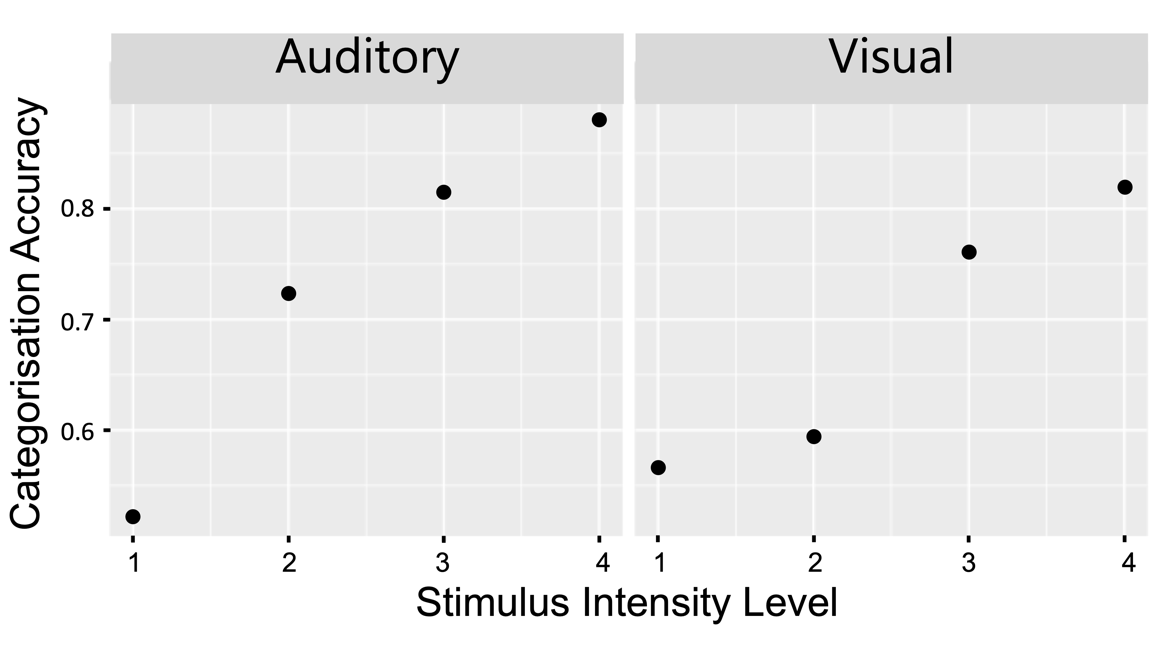


**Fig A. Control Experiment: Categorisation Accuracy as a Function of Stimulus Intensity.** Categorisation accuracy in the control experiment increased approximately linearly with increasing stimulus intensity in the auditory task as in the visual task. Means are plotted for the same 4 participants who completed the main experiment and the control experiment.

***Model Specification***

All models were the same as in the main study, however, as the orientation-dependent noise parameter, $\psi$, did not reliably improve model fits (see **Table 2**) and did not affect the conclusions, we did not include it in any variations of the models in the control study.

***Results***

In line with the results from the main experiment, the model-based analysis found that the best performing model for all subjects was one from the scaled evidence strength class (25% for linear, 50% for quadratic and 25% for free-exponent). This preference for the scaled evidence strength models was consistent with the summed *AIC* and *BIC* values which were the lowest for the free-exponent and quadratic models, respectively (see **Table A** and **Fig B**). As in the main experiment, we also wanted to determine if different parameter settings were required to account for the data across modalities. We used the same participants’ data from the visual task from the main experiment and found that the flexible settings model was the best performing model (see **Table B**) on average, according to the summed *AIC* and *BIC* scores. At the individual level, for all subjects the best performing model was the flexible settings model (see ‘Parameter Settings of the Free-Exponent Models Across Modalities’ in Model Specification for model descriptions). We, therefore, replicated the same set of effects observed in the main experiment with the data from the control experiment.

**Table A**

***Model Fits for Control Data***

| Model | Different SDs Auditory Task | | |
| --- | --- | --- | --- |
|  | *AIC_sum_* | *BIC_sum_* | Preferred Model^a^ |
| Fixed | 8923.07 | 9216.05 | 0 |
| Linear | 7167.80 | 7647.23 | 1 |
| Quadratic | 7026.60 | **7506.03** | 2 |
| Free-Exponent | **7023.76** | 7529.82 | 1 |
| LPPR | 10265.82 | 10558.80 | 0 |
| LPPR + D noise | 9100.79 | 9420.41 | 0 |
| LPPR: Free Prior | 10359.60 | 10705.85 | 0 |

*Note.* LPPR refers to the log posterior probability ratio models, D noise refers to models with a decision noise parameter. ^a^The number of participants for whom the model was the best fitting model. Where there was inconsistency between *AIC* and *BIC* for the best fitting model, we chose the model with the lowest *AIC,* based on the results of the model recovery. Bolded values indicate best performing model according to a given metric.

**Table B**

***Parameter Settings Across Modalities for Control Data***

| Model | *AIC_sum_* | *BIC_sum_* | Preferred Model^a^ |
| --- | --- | --- | --- |
|  | Different Means Task | | |
| Common Settings | 15339.39 | 15845.45 | 0 |
| Different Noise Settings | 15121.19 | 15733.79 | 0 |
| Flexible Settings | **14512.13** | **15524.25** | **4** |

*Note.* LPPR refers to the log posterior probability ratio models, D noise refers to models with a decision noise parameter. ^a^The number of participants for whom the model was the best fitting model. Where there was inconsistency between *AIC* and *BIC* for the best fitting model, we chose the model with the lowest *AIC,* based on the results of the model recovery. Bolded values indicate best performing model according to a given metric.


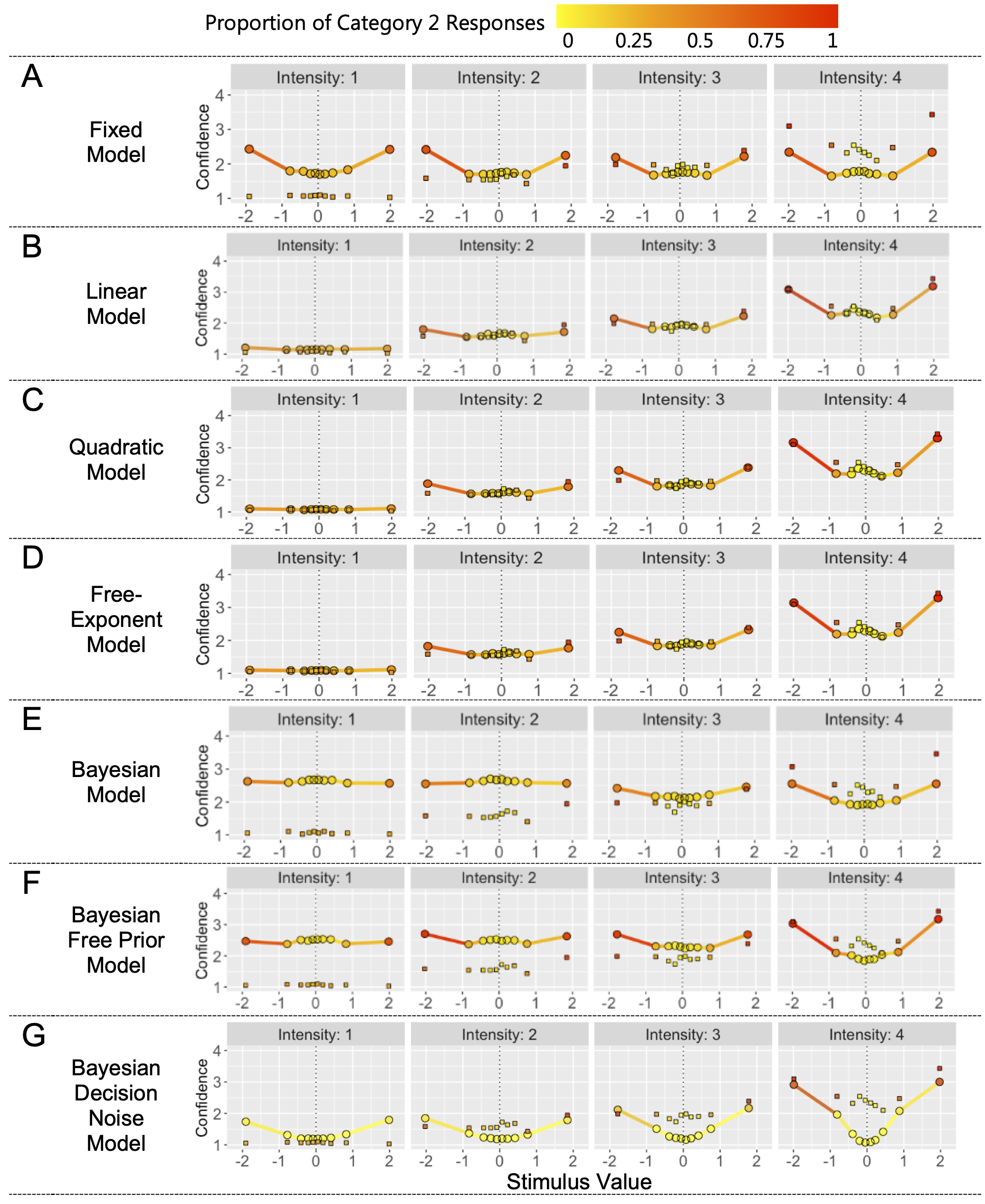


**Fig B. Model Comparison for Control Study.** In all plots, mean confidence (y axis) and proportion of Category 2 responses (colour) for binned standardised stimulus values (x axis). Square data points show means for experimental data and solid lines and circular data points show means for model predictions.
