# Supplementary material for: Modality independent or modality specific? Common computations underlie confidence judgements in visual and auditory decisions
Source: PLoS Comput Biol. 2023 Jul 14;19(7):e1011245. doi: 10.1371/journal.pcbi.1011245 (PMC10426961; doi:10.1371/journal.pcbi.1011245)
Supplement: S2 Text — Table A. Category and Confidence Model-Free GLMMs With Data From All Tasks and Modalities. Fig A. Category and Confidence Model-Free GLMMs With Data From All Tasks and Modalities (DOCX) [file pcbi.1011245.s002.docx]

**S2 Text: Secondary Model-Free GLMMs**

The model free GLMMs shown in **Fig 3** confirmed that variations in evidence strength and stimulus intensity had the expected effect on category and confidence responses across tasks and modalities. In this analysis, the GLMMs were fit to data from each task and modality separately because the goal was to qualitatively compare the behavioural effects of the relevant stimulus manipulations across tasks and modalities, without imposing any assumptions about the similarity of these effects. To further investigate similarities in the category and confidence response profiles across modalities, we therefore conducted a secondary analysis. We fit GLMMs to the evidence and intensity data from all tasks and modalities concurrently to investigate if the data could be described by the same set of effects or alternatively, if there were interactions between the effect of certain stimulus manipulations and the different domains.

We fit one model to the category response data and a second model to the confidence response data. We included a task and modality indicator variable as a predictor in each GLMM. In this analysis, we focused more on interactions between evidence (evidence for category 2 and category diagnosticity; see **Results**), intensity and modality, rather than tasks, as our main empirical interest was in similarities in category/confidence response profiles across sensory modalities. Furthermore, we a priori expected some differences in response profiles across tasks, as the distributions of evidence values were inherently different from the different category structures (see **Fig 2B**). We report the fixed effects, 95% confidence intervals and significance values for each GLMM in **Table A**. In the following sections, we provide a summary of significant effects in text; associated statistical tests can be found in the table.

Table A

Category and Confidence Model-Free GLMMs With Data From All Tasks and Modalities

| Predictors | Estimate | 95% CI | *p*-value |
| --- | --- | --- | --- |
|  | Category | | |
| (Intercept) | 0.73 | 0.49  –  1.10 | 0.134 |
| Intensity | 1.25 | 0.75  –  2.09 | 0.388 |
| Evidence | 2.40*** | 1.72  –  3.36 | <0.001 |
| Task^a^ | 1.31** | 1.08  –  1.59 | 0.007 |
| Modality^b^ | 1.05 | 0.61  –  1.79 | 0.870 |
| Intensity × Evidence | 2.28*** | 1.63  –  3.21 | <0.001 |
| Intensity × Task | 1.48 | 0.92  –  2.38 | 0.105 |
| Evidence × Task | 1.75*** | 1.42  –  2.16 | <0.001 |
| Intensity × Modality | 1.61 | 0.87  –  2.95 | 0.128 |
| Evidence × Modality | 1.60 | 1.00  –  2.56 | 0.052 |
| Task × Modality | 0.73 | 0.47  –  1.14 | 0.163 |
| Intensity × Evidence × Task | 1.96*** | 1.44  –  2.68 | <0.001 |
| Intensity × Evidence × Modality | 1.38 | 0.93  –  2.04 | 0.108 |
| Intensity × Task × Modality | 0.48* | 0.25  –  0.91 | 0.024 |
| Evidence × Task × Modality | 0.97 | 0.62  –  1.51 | 0.892 |
| Evidence × Intensity × Task × Modality | 0.71 | 0.43  –  1.19 | 0.195 |
|  | Confidence | | |
| (Intercept) | 1.82*** | 1.66  –  1.99 | <0.001 |
| Intensity | 0.62*** | 0.45  –  0.80 | <0.001 |
| Evidence | 0.13*** | 0.07  –  0.19 | <0.001 |
| Task^a^ | 0.16*** | 0.10  –  0.22 | <0.001 |
| Modality^b^ | 0.06 | -0.25  –  0.38 | 0.705 |
| Intensity × Evidence | 0.10*** | 0.05  –  0.15 | <0.001 |
| Intensity × Task | 0.10* | 0.01  –  0.19 | 0.026 |
| Evidence × Task | 0.00 | -0.04  –  0.04 | 0.851 |
| Intensity × Modality | -0.24** | -0.38  – -0.10 | 0.001 |
| Evidence × Modality | 0.02 | -0.06  –  0.09 | 0.671 |
| Task × Modality | -0.23* | -0.40  – -0.05 | 0.015 |
| Intensity × Evidence × Task | 0.00 | -0.03  –  0.04 | 0.814 |
| Intensity × Evidence × Modality | 0.00 | -0.06  –  0.06 | 0.960 |
| Intensity × Task × Modality | -0.08 | -0.19  –  0.02 | 0.125 |
| Evidence × Task × Modality | 0.04 | -0.02  –  0.11 | 0.198 |
| Evidence × Intensity × Task × Modality | 0.04 | -0.02  –  0.11 | 0.200 |

*Note.* CI terms refer to 95% confidence intervals calculated using the profile likelihood method. Significance values are obtained using the Satterthwaite approximation to calculate the degrees of freedom for the t-distribution based on the estimated variance-covariance matrix of the model parameters [1]. For category GLMMs (top section), we report the odds ratio (exponentiated coefficient estimate) for ease of interpretation. The odds ratio represents the change in odds of the outcome for a one-unit change in the predictor variable. For confidence GLMMs (bottom section), we report standardised regression coefficients.

**p* < 0.050, ***p* < 0.010, ****p* < 0.001

^a^Different SDs task coded as 0 and different means task coded as 1.

^b^Auditory modality coded as 0 and visual modality coded as 1.

***Category Responses***

For category responses, we fit linear mixed models using a logistic link function to predict category responses from stimulus intensity, evidence for category 2 (see **Equation 1**), modality (visual or auditory), task (different means or different SDs) and all possible interactions between these predictor variables.

**Main Effects.** As expected, we found a significant effect of evidence, such that for a one-unit increase in category 2 evidence, the odds of a participant giving a category 2 response increased by a factor of 2.4 (see **Table A**). We also found a significant effect of task, such that the odds of a participant giving a category 2 response was 1.31 times higher for the different means task compared to the different SDs task.^^[[1]](#footnote-1)^^ We did not observe a main effect of intensity or modality on category 2 responses.

**Interactions.** As expected, we found a significant intensity × evidence interaction where, the effect of evidence on category responses was modulated by stimulus intensity. As shown in **Fig A** **(A)**, we observed the strongest effect of evidence on confidence at the highest intensity levels.

We also found a significant evidence × task interaction and a significant evidence × intensity × task interaction, suggesting that the effect of evidence and the evidence × intensity interaction depended on the task type (different means or different SDs). As described above, any interactive effect between the evidence and task variables was not surprising given the different category structures and the differences in relative changes in evidence for category 2 across stimulus values (see **Fig 2B**). Thus, to summarise the interactions described above, we confirmed that participants’ category responses aligned with evidence strength but this relationship was modulated by stimulus intensity and task type (see **Fig 3B**).

Interestingly, we found a significant intensity × task × modality interaction, suggesting that the effect of intensity on category responses depended on both task type and modality. Further investigation of this effect showed that, as shown in **Fig A** **(C),** participants had a notable category 1 bias which reduced with stimulus intensity. This relationship was not as clear for the auditory different SDs task, where we observed an increase in the category 1 bias at the highest intensity level.

Overall, these findings provide converging evidence that participants used a similar strategy for their category choices across tasks and modalities. We did, however, observe several intensity-based and task-based interactions, providing some exceptions to the similarity in effects. The former set of interactions were likely driven by the fact that participants did not appear to be able to distinguish between the two lowest intensity levels and the two highest intensity levels in the auditory modality. We provide additional discussion of this finding in **S1 Text**.


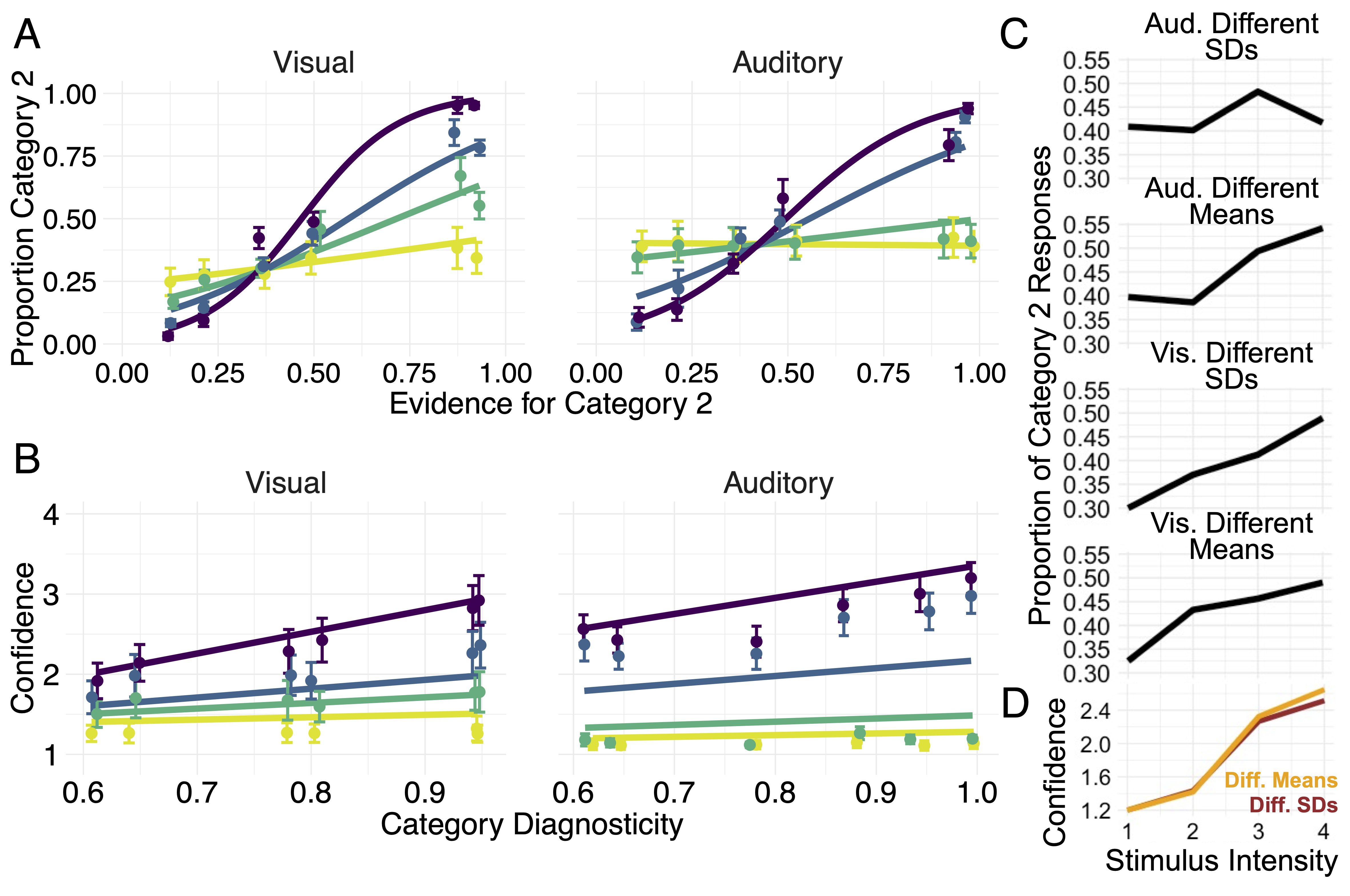


**Fig A. Category and Confidence Model-Free GLMMs With Data From All Tasks and Modalities.** (A) Category decisions as a function of category 2 evidence. Data points show empirical data from visual modality (left) and auditory modality (right). Solid lines show logistic model predictions for the fixed effect of evidence at each intensity level. (B) Model-free GLMM. Confidence as a function of category diagnosticity. Data points show empirical data from visual modality (left) and auditory modality (right). Solid lines show linear model predictions for the fixed effect of category diagnosticity at each intensity level. (C) Proportion of category 2 responses as a function of stimulus intensity. Each panel shows a different domain. Solid lines show empirical data. (D) Confidence as a function of stimulus intensity. Different means task shown in orange and different SDs task shown in brown. Solid lines show empirical data. Error bars show ± 1 *SEM*.

***Confidence Responses***

For confidence responses, we fit linear mixed models using a linear function to predict category responses from stimulus intensity, category diagnosticity (see **Equation 2**), modality (visual or auditory), task (different means or different SDs) and the interactions among these predictors.

**Main Effects.** As expected, we found a significant effect of intensity and evidence, where confidence increased with both stimulus intensity and evidence (category diagnosticity), see **Fig A (B)**. We found a significant effect of task, with overall greater confidence in the different means task (*M* = 1.90, *SD* = 1.05) compared to the different SDs task (*M* = 1.85, *SD* = 1.01). We did not find a significant effect of modality on confidence.

**Interactions.** As expected, we found a significant evidence × intensity interaction, suggesting that the strength of the effect of evidence on confidence depended on stimulus intensity. As shown in **Fig A (B)**, we observed the strongest effect of evidence on confidence at the highest intensity levels.

We found a significant intensity × modality interaction, suggesting that the effect of stimulus intensity on confidence depended on the modality. This interaction was not surprising, given the different confidence response profiles across intensity levels in the visual and auditory task (see **S1 Text** for further discussion of this issue). We also found a significant intensity × task interaction, suggesting that the effect of intensity depended on the task. This interaction was surprising and further inspection (see **Fig A (D)**) suggests that the effect was driven by a difference in confidence across tasks for the highest intensity levels, where confidence was greater in the different means task.

Overall, these findings suggest that participants’ confidence judgements account for basic stimulus features in a similar way across modalities. The exception being, as with the category response data, that participants did not appear to distinguish between the two lowest intensity levels and the two highest intensity levels in the auditory modality. We, therefore, observed several intensity-based interactions. The general patterns are nonetheless comparable across modalities and tasks and provide converging evidence that the computations used for confidence are the same across domains.

**References**

1. Lüdecke D. _sjPlot: Data Visualization for Statistics in Social Science_. R package version 2.8.12. 2022. Available: <https://CRAN.R-project.org/package=sjPlot>

1. Although we observed a notable category 1 bias in both tasks. [↑](#footnote-ref-1)
