# Supplementary material for: Modality independent or modality specific? Common computations underlie confidence judgements in visual and auditory decisions
Source: PLoS Comput Biol. 2023 Jul 14;19(7):e1011245. doi: 10.1371/journal.pcbi.1011245 (PMC10426961; doi:10.1371/journal.pcbi.1011245)
Supplement: S3 Text — Fig A. Unscaled-Evidence Strength Model: The Distance Model. Fig B. Scaled-Evidence Strength Model: The Linear Model. Fig C. Scaled-Evidence Strength Model: The Quadratic Model. Fig D. Scaled-Evidence Strength Model: The Free-Exponent Model with Orientation Dependent Noise. Fig E. Bayesian Model: Log Posterior Probability Ratio. Fig F. Bayesian Model: Log Posterior Probability Ratio with Orientation Dependent Noise. Fig G. Bayesian Model: Log Posterior Probability Ratio with Decision Noise. Fig H. Bayesian Model: Log Posterior Probability Ratio with Free Category Distributions Parameters. Fig I. Parameter Settings Across Modalities: Different Means Task. Fig J. Parameter Settings Across Modalities: Different SDs Task (DOCX) [file pcbi.1011245.s003.docx]

**S3 Text: Alternative Visualisation of Model Fits from Main Experiment**

**Fig A - Fig J** show the data and model predictions from the main experiment. These visualisations are an alternative to the plots shown in **Fig 5**, where we calculate mean combined category and confidence response per bin which averages over category and confidence. In the figures below, we do not collapse over category and confidence and instead show confidence on the y axes and use colour to show proportion of Category 2 responses. See figure labels for more details.

**
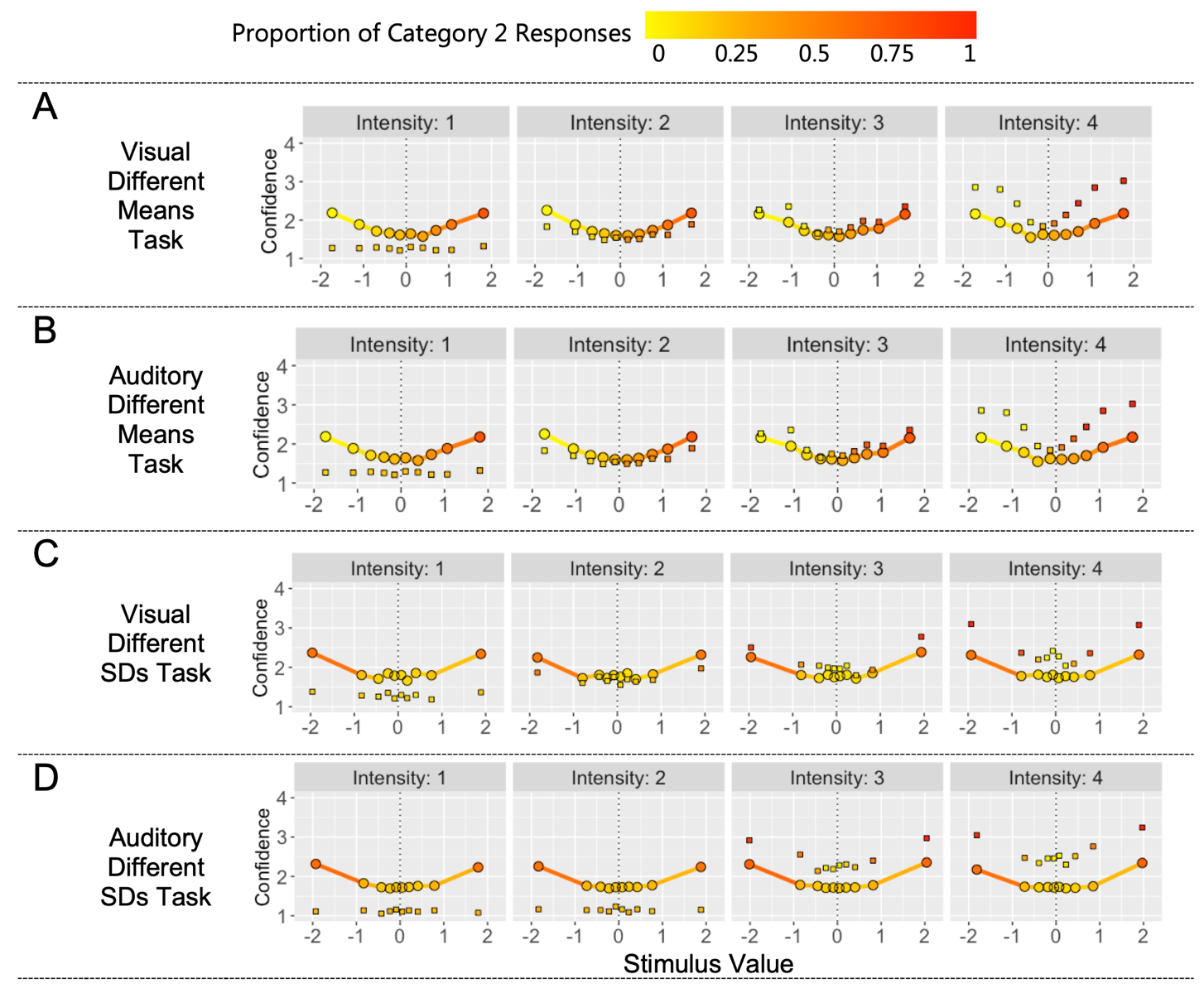
**

**Fig A. Unscaled-Evidence Strength Model: The Distance Model.** In all plots, mean confidence (y axis) and proportion of Category 2 responses (colour) for binned standardised stimulus values (x axis). Square data points show means for experimental data and solid lines and circular data points show means for model predictions. Model was fit to data from each task and modality separately. Model predictions displayed for: (A) visual different means task, (B) auditory different means task, (C) visual different SDs task and (D) auditory different SDs task.

***
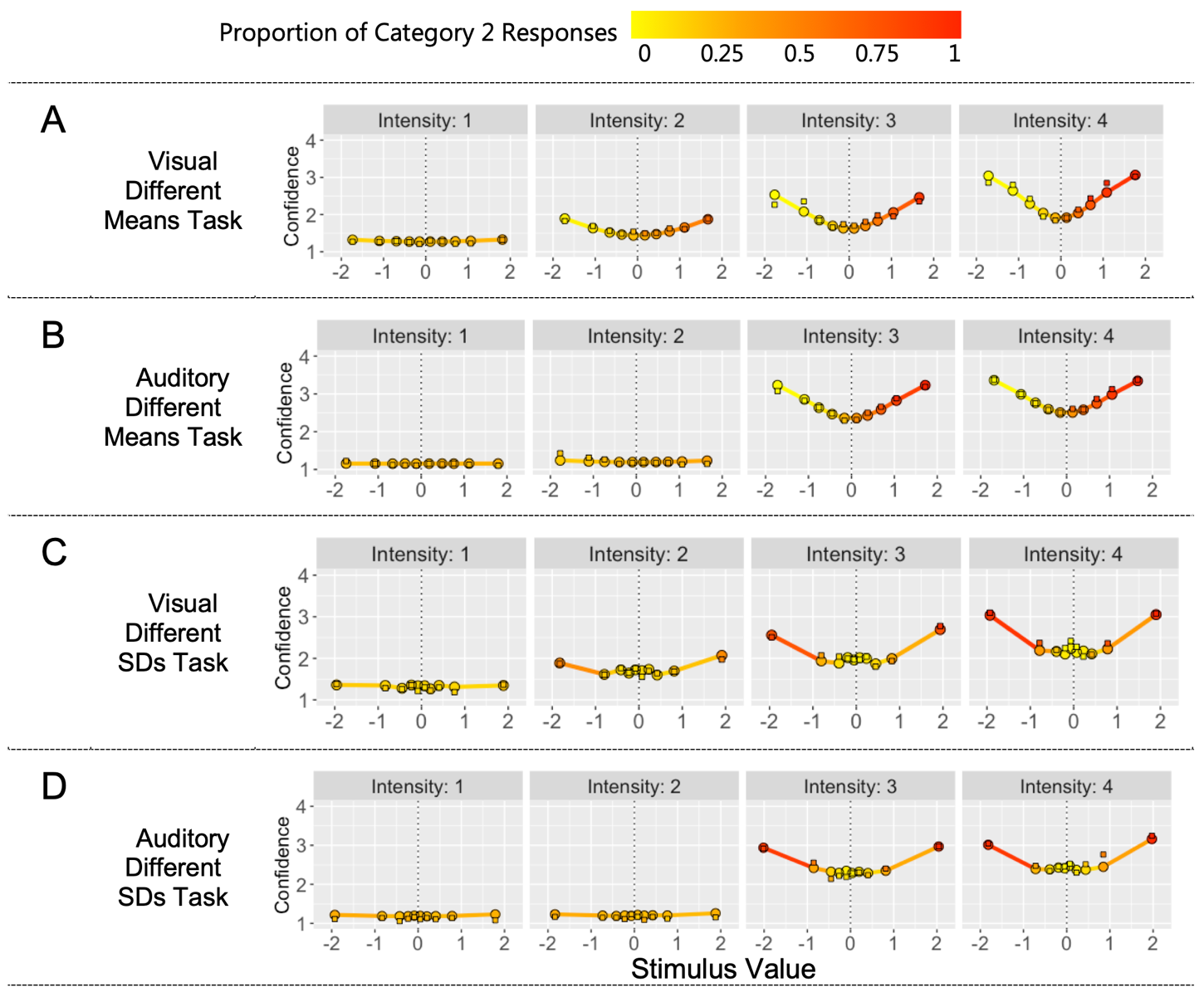
***

**Fig B. Scaled-Evidence Strength Model: The Linear Model.** In all plots, mean confidence (y axis) and proportion of Category 2 responses (colour) for binned standardised stimulus values (x axis). Square data points show means for experimental data and solid lines and circular data points show means for model predictions. Model was fit to data from each task and modality separately. Model predictions displayed for: (A) visual different means task, (B) auditory different means task, (C) visual different SDs task and (D) auditory different SDs task.

***
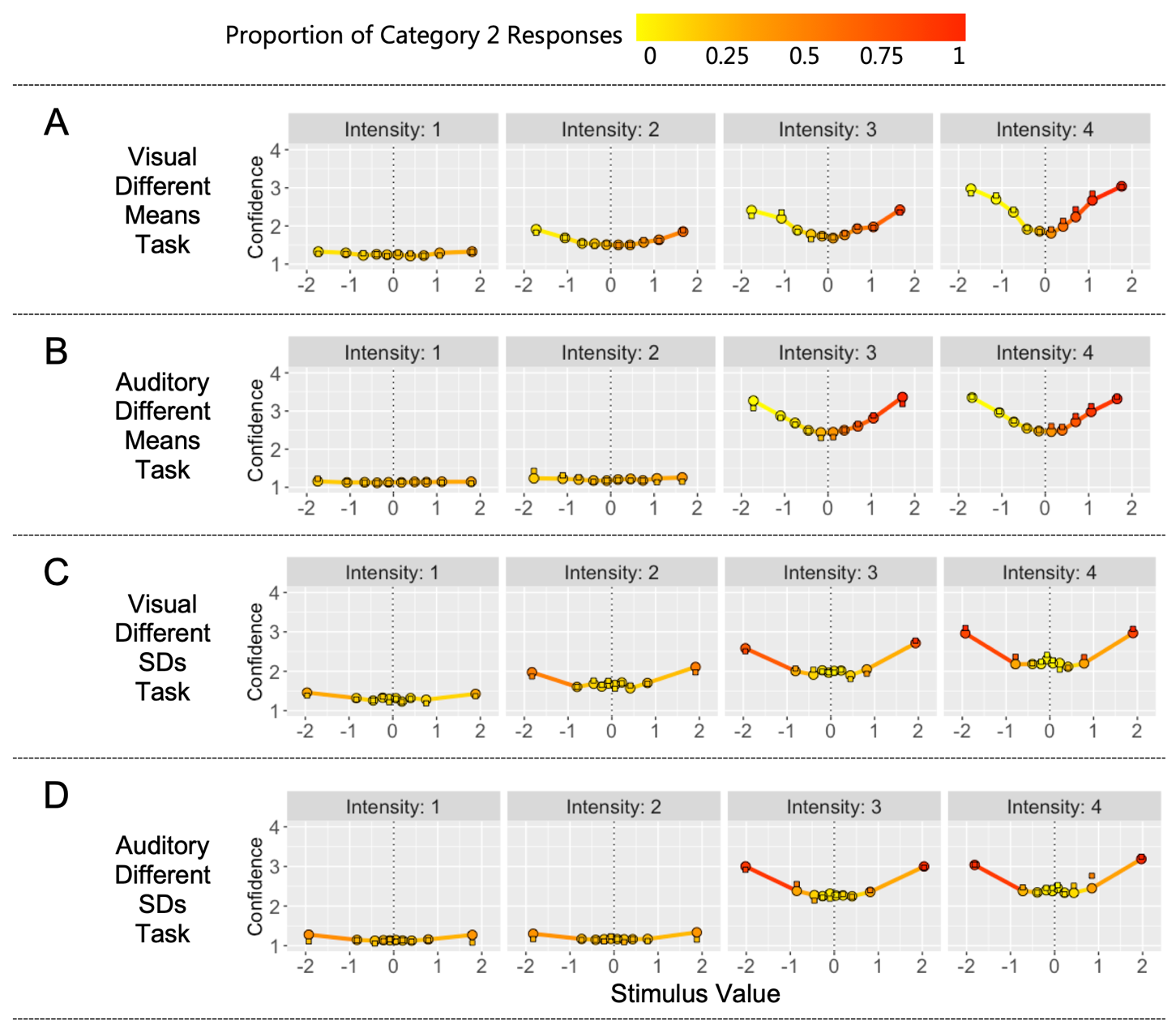
***

**Fig C. Scaled-Evidence Strength Model: The Quadratic Model.** In all plots, mean confidence (y axis) and proportion of Cat 2 responses (colour) for binned standardised stimulus values (x axis). Square data points show means for experimental data and solid lines and circular data points show means for model predictions. Model was fit to data from each task and modality separately. Model predictions displayed for: (A) visual different means task, (B) auditory different means task, (C) visual different SDs task and (D) auditory different SDs task.

***
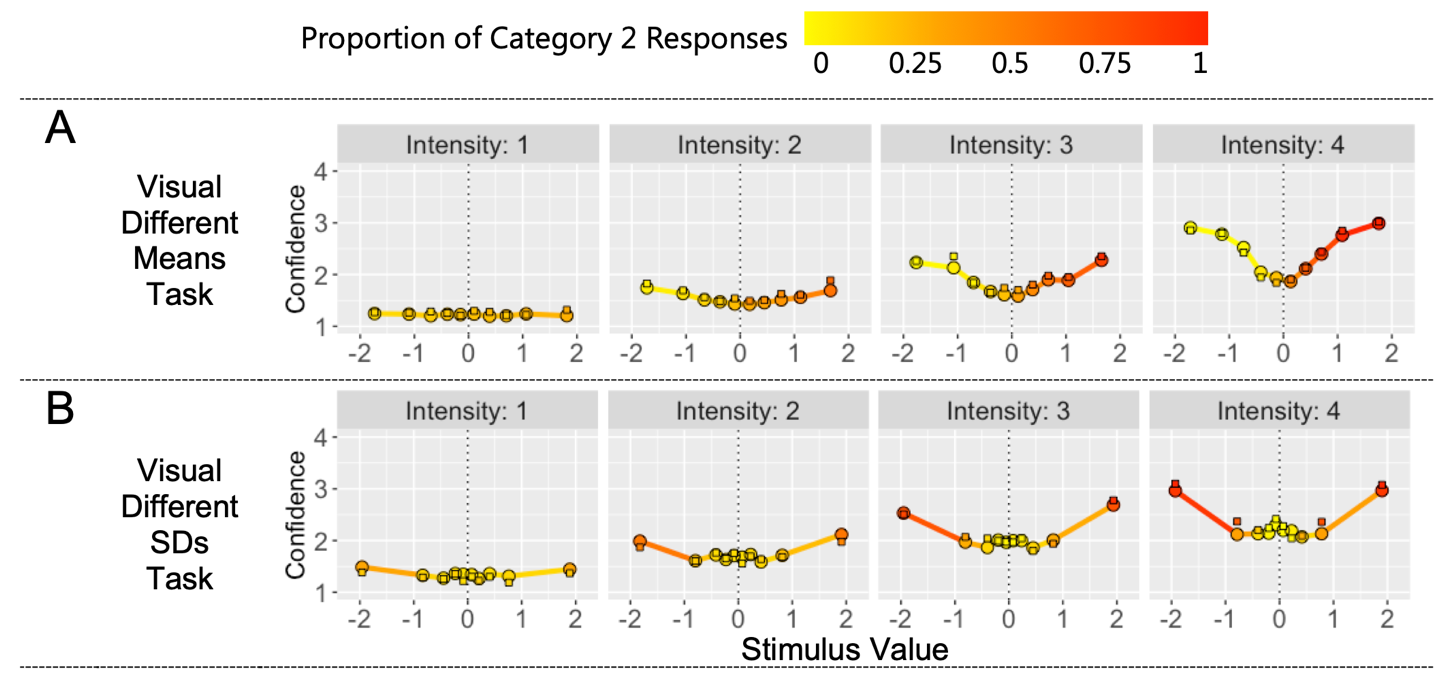
***

**Fig D. Scaled-Evidence Strength Model: The Free-Exponent Model with Orientation Dependent Noise.** In all plots, mean confidence (y axis) and proportion of Category 2 responses (colour) for binned standardised stimulus values (x axis). Square data points show means for experimental data and solid lines and circular data points show means for model predictions. Model was fit to data from each task. Model predictions displayed for: (A) visual different means task and (B) visual different SDs task.

***
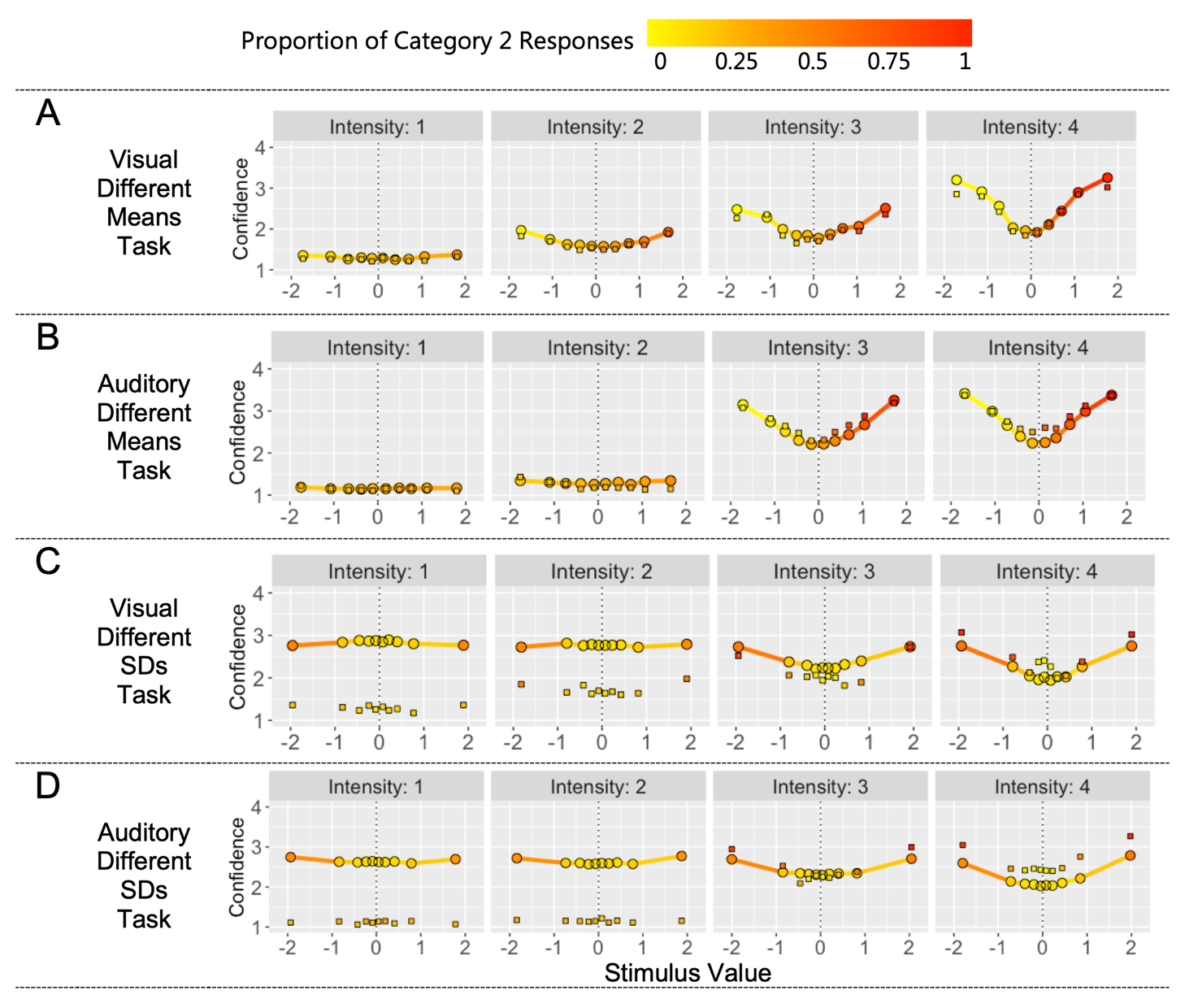
***

**Fig E. Bayesian Model: Log Posterior Probability Ratio.** In all plots, mean confidence (y axis) and proportion of Category 2 responses (colour) for binned standardised stimulus values (x axis). Square data points show means for experimental data and solid lines and circular data points show means for model predictions. Model was fit to data from each task and modality separately. Model predictions displayed for: (A) visual different means task, (B) auditory different means task, (C) visual different SDs task and (D) auditory different SDs task.

***
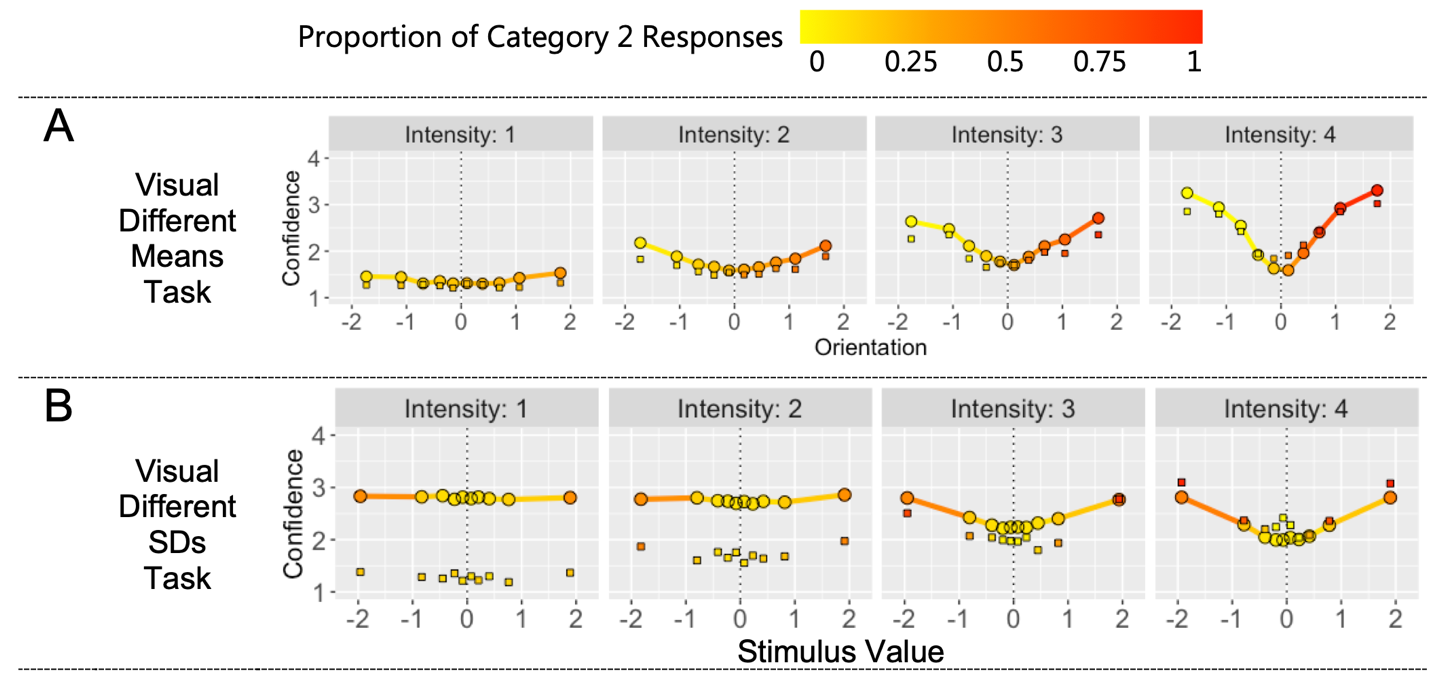
***

**Fig F. Bayesian Model: Log Posterior Probability Ratio with Orientation Dependent Noise.** In all plots, mean confidence (y axis) and proportion of Category 2 responses (colour) for binned standardised stimulus values (x axis). Square data points show means for experimental data and solid lines and circular data points show means for model predictions. Model was fit to data from each task. Model predictions displayed for: (A) visual different means task and (B) visual different SDs task.

***
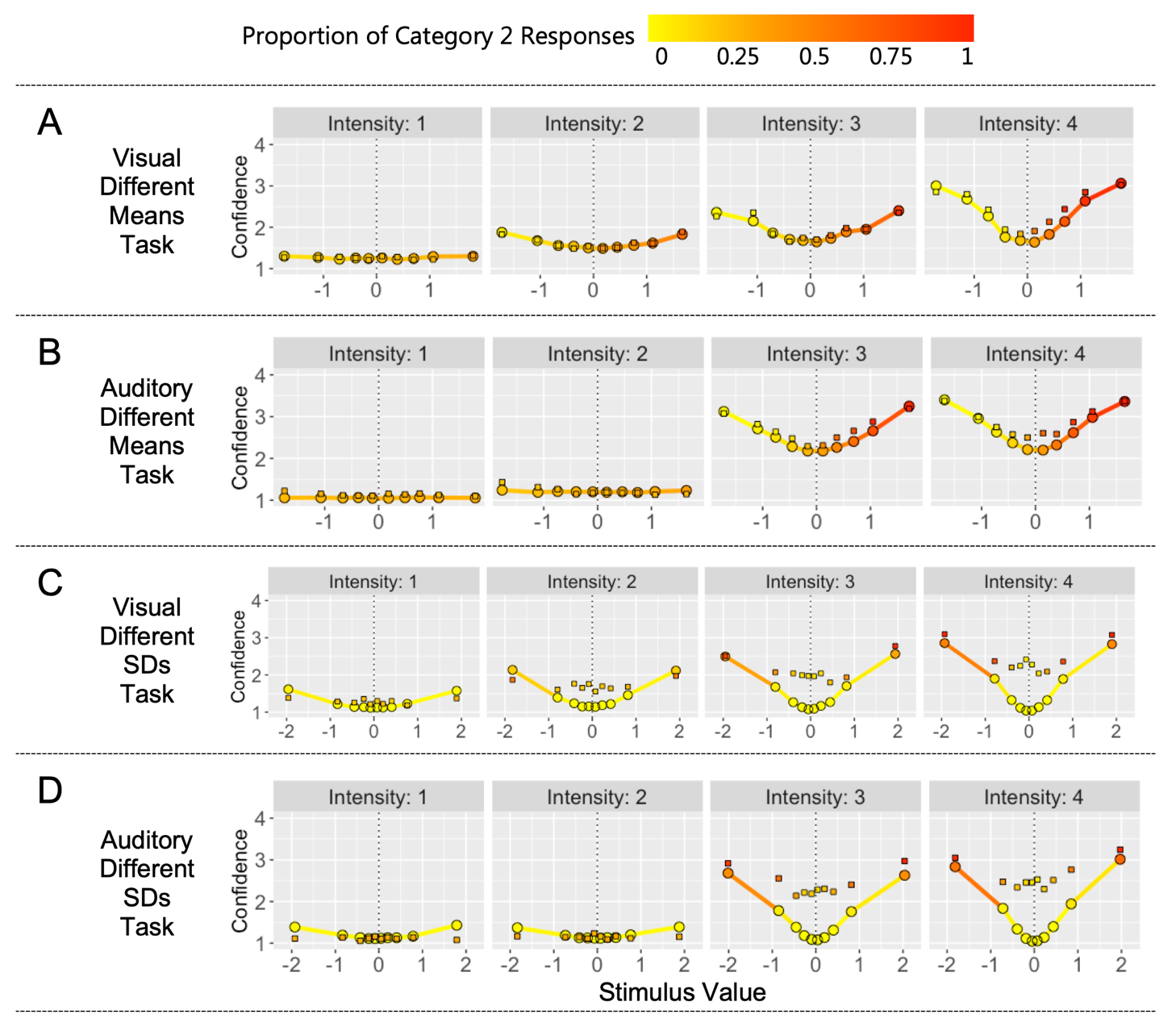
***

**Fig G. Bayesian Model: Log Posterior Probability Ratio with Decision Noise.** In all plots, mean confidence (y axis) and proportion of Cat 2 responses (colour) for binned standardised stimulus values (x axis). Square data points show means for experimental data and solid lines and circular data points show means for model predictions. Model was fit to data from each task and modality separately. Model predictions displayed for: (A) visual different means task, (B) auditory different means task, (C) visual different SDs task and (D) auditory different SDs task.

***
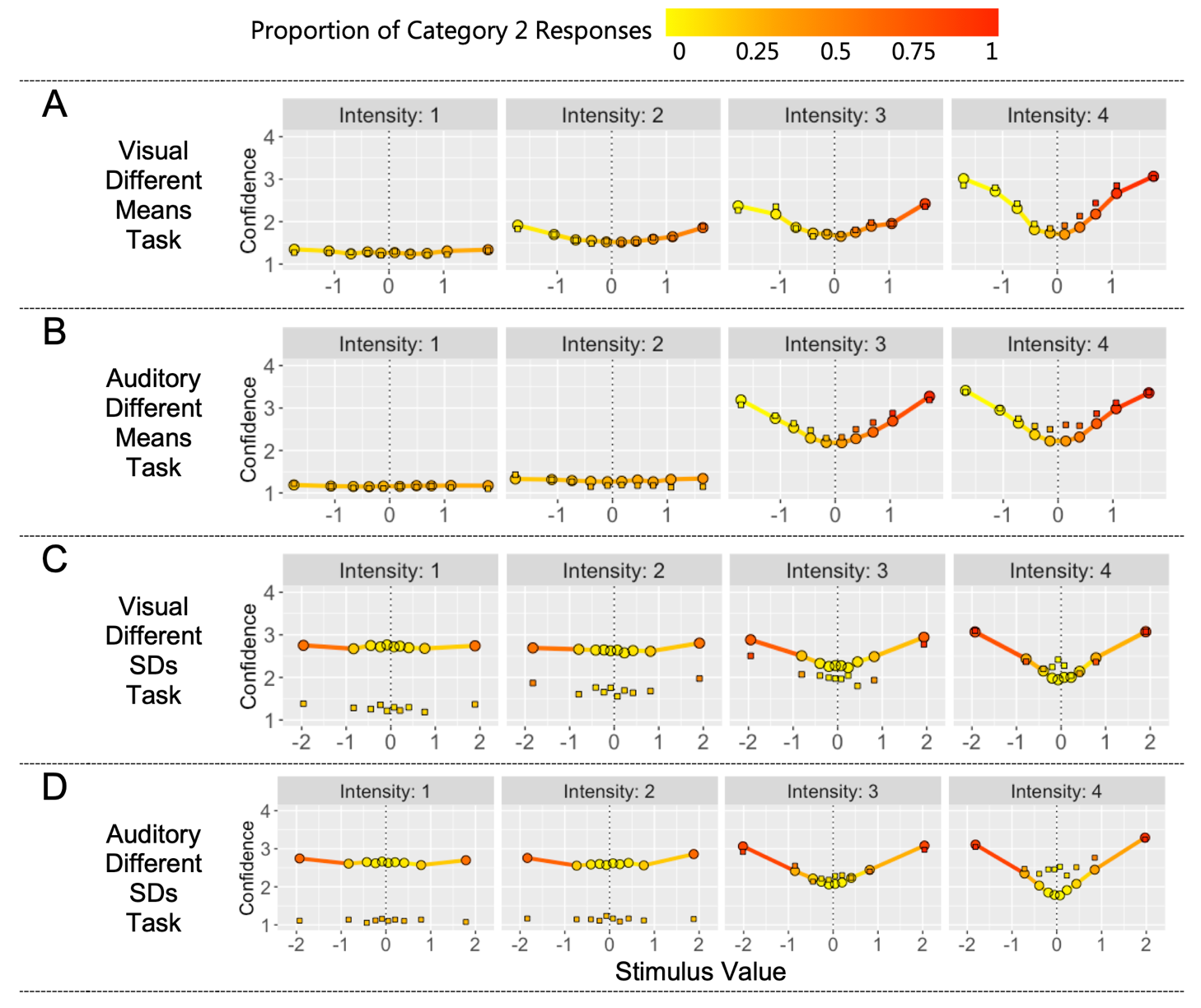
***

**Fig H. Bayesian Model: Log Posterior Probability Ratio with Free Category Distributions Parameters.** In all plots, mean confidence (y axis) and proportion of Cat 2 responses (colour) for binned standardised stimulus values (x axis). Square data points show means for experimental data and solid lines and circular data points show means for model predictions. Model was fit to data from each task and modality separately. Model predictions displayed for: (A) visual different means task, (B) auditory different means task, (C) visual different SDs task and (D) auditory different SDs task.


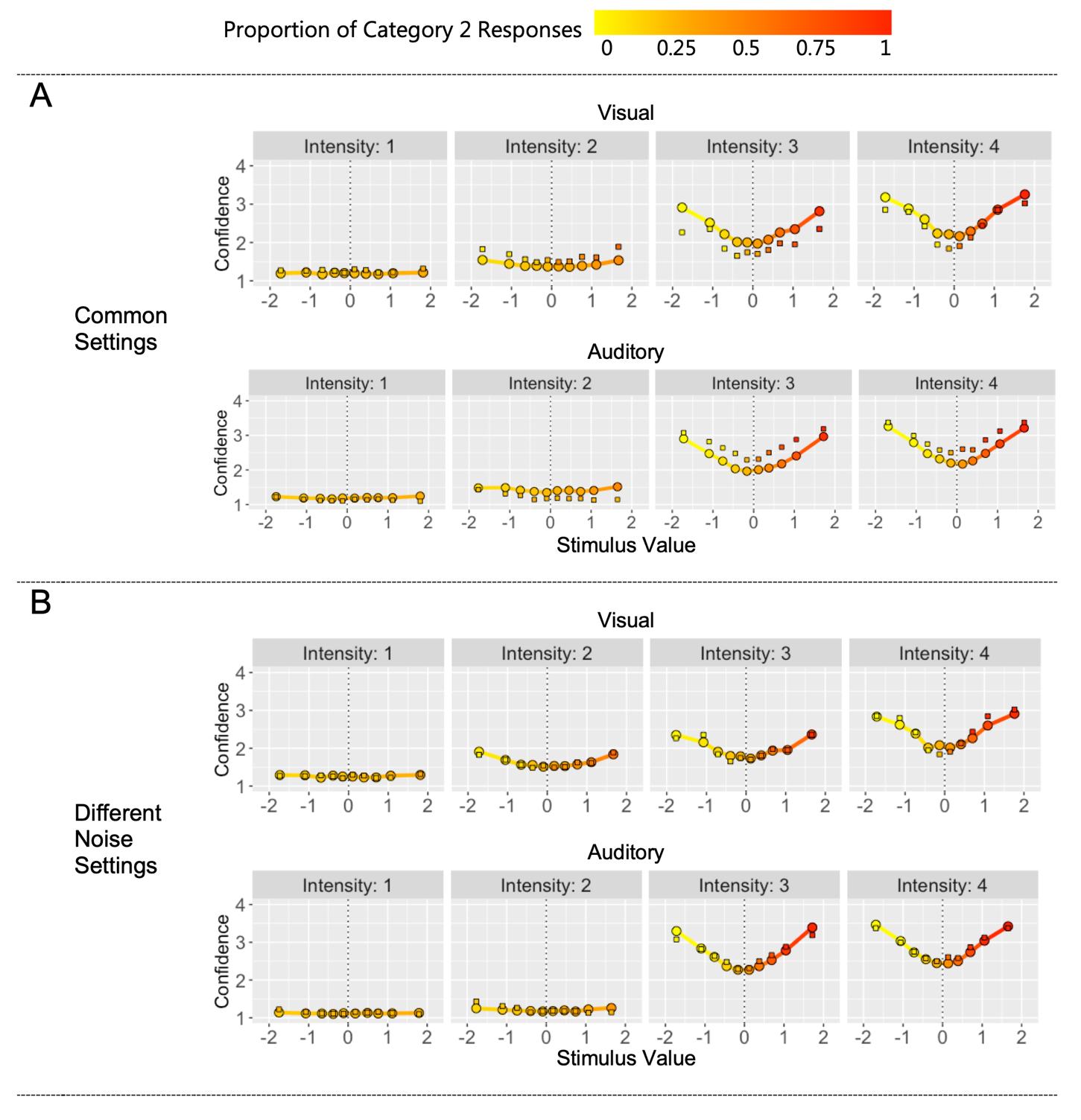


**Fig I. Parameter Settings Across Modalities: Different Means Task.** In all plots, mean confidence (y axis) and proportion of Category 2 responses (colour) for binned standardised stimulus values (x axis). Square data points show means for experimental data and solid lines and circular data points show means for model predictions from (A) the common settings model and (B) the different noise settings model.


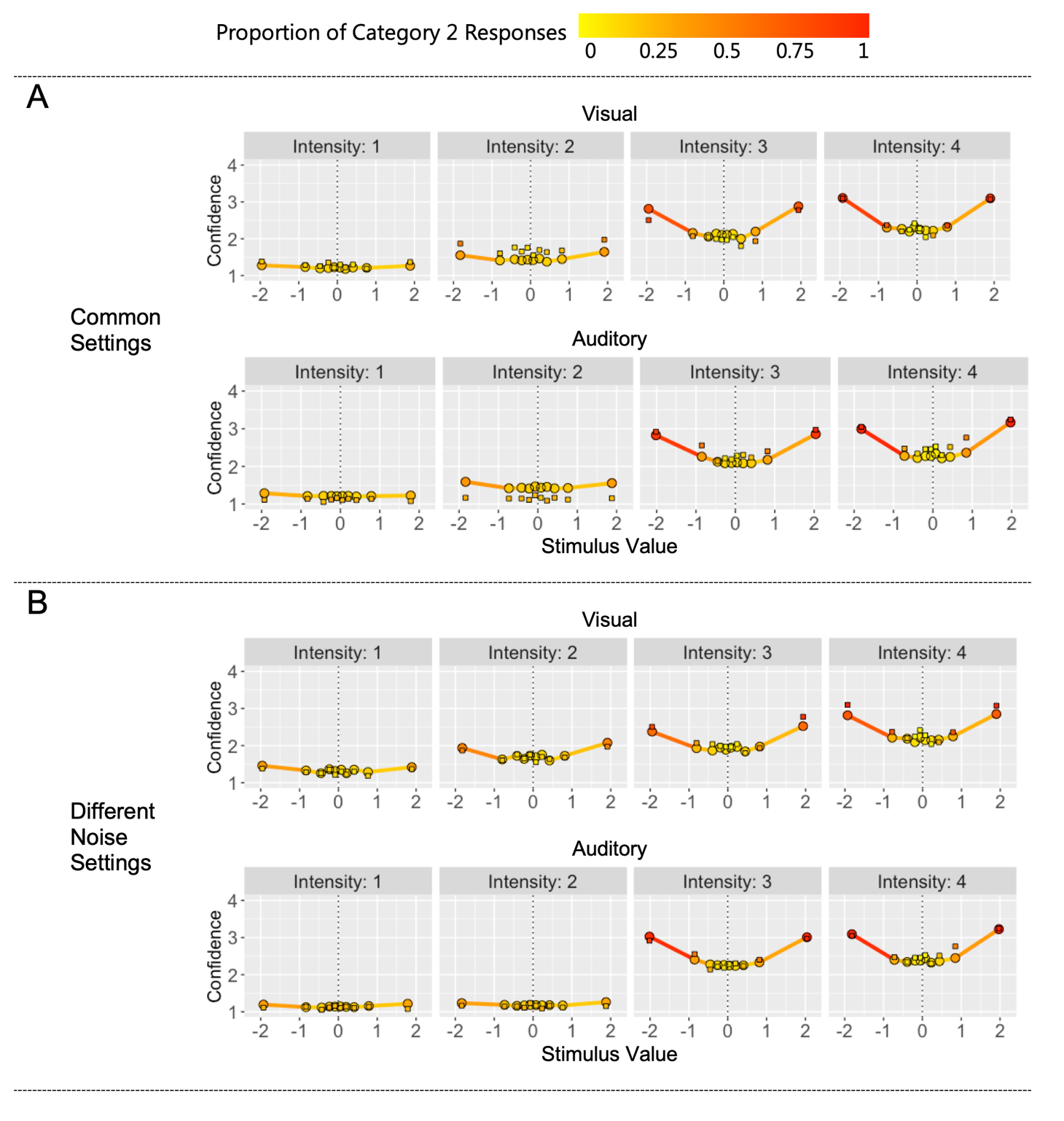


**Fig J. Parameter Settings Across Modalities: Different SDs Task.** In all plots, mean confidence (y axis) and proportion of Category 2 responses (colour) for binned standardised stimulus values (x axis). Square data points show means for experimental data and solid lines and circular data points show means for model predictions from (A) the common settings model and (B) the different noise settings model.
