# Supplementary material for: Modality independent or modality specific? Common computations underlie confidence judgements in visual and auditory decisions
Source: PLoS Comput Biol. 2023 Jul 14;19(7):e1011245. doi: 10.1371/journal.pcbi.1011245 (PMC10426961; doi:10.1371/journal.pcbi.1011245)
Supplement: S4 Text — Fig A. Model Recovery for Core Models. Fig B. Model Recovery for Models Used To Compare Parameter Settings Across Modalities (DOCX) [file pcbi.1011245.s004.docx]

**S4 Text: Model Recovery**

We explored the model recovery properties of the core models: distance, linear, quadratic, free-exponent, log posterior probability ratio (labelled as *Bayesian* in the Figures below) and log posterior probability ratio with free category distribution parameters (labelled as *Bayesian Free Prior*) for the different SDs task. Although the data were, on average, best fit by the model used to generate that data, some of the models were harder to distinguish than others (see **Fig A**). As expected, there was a high degree of confusability for the linear, quadratic, and free-exponent models. This result was expected because the free-exponent model contained both the linear and quadratic models as special cases. In particular, simulated exponent parameters were constrained between 1 and 4 and therefore, there was a high degree of similarity in model behaviour between these models, especially for the free-exponent and quadratic models. As shown in **Fig A**, there was also some confusability among the Bayesian models. This was also expected because the log posterior probability ratio model with free category distribution parameters contained the log posterior probability ratio model as a special case, that is, where the estimated category distribution parameters matched the true category distribution parameters.

Importantly, we did not find much confusability across the model classes, where the unscaled evidence strength model (Distance), scaled evidence strength models (Linear, Quadratic, Free-Exponent) and Bayesian (log posterior probability ratio, log posterior probability ratio with free category distribution parameters) were distinguishable from one another using both *AIC* (**Fig A** **(A)**) and *BIC* (**Fig A** **(B)**) for model selection. Of note, the models appeared to recover better on average when using *AIC* for model selection and therefore, where there were any differences in the best-fitting model during model comparison, we selected the model with the lowest *AIC*.


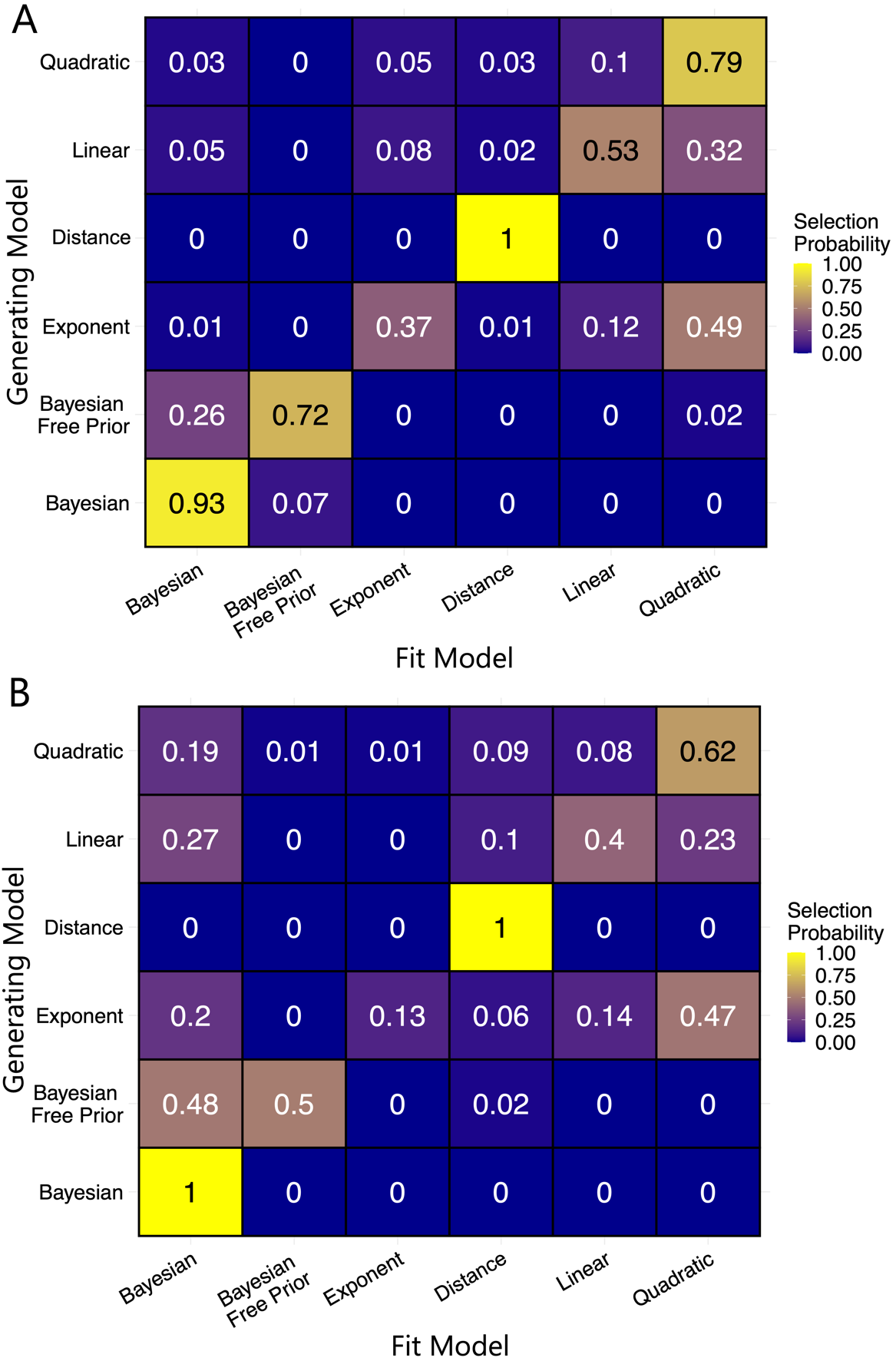


**Fig A. Model Recovery for Core Models.** Numbers and colours denote the probability that the data generated with model X are best fit by model Y. (A) Selection probabilities calculated using *AIC* for model selection. (B) Selection probabilities calculated using *BIC* for model selection.

We also performed a model recovery for the models used to compare parameter settings across modalities. As shown in **Fig B**, we found that the simulated data were almost always best fit by the model used to generate that data. This result held for both *AIC* (**Fig B (A)**) and *BIC* (**Fig B (B)**). This model recovery validated the model comparison results that were used to compare the parameter settings of the free-exponent model across the visual and auditory modalities (see **Figure 7**).


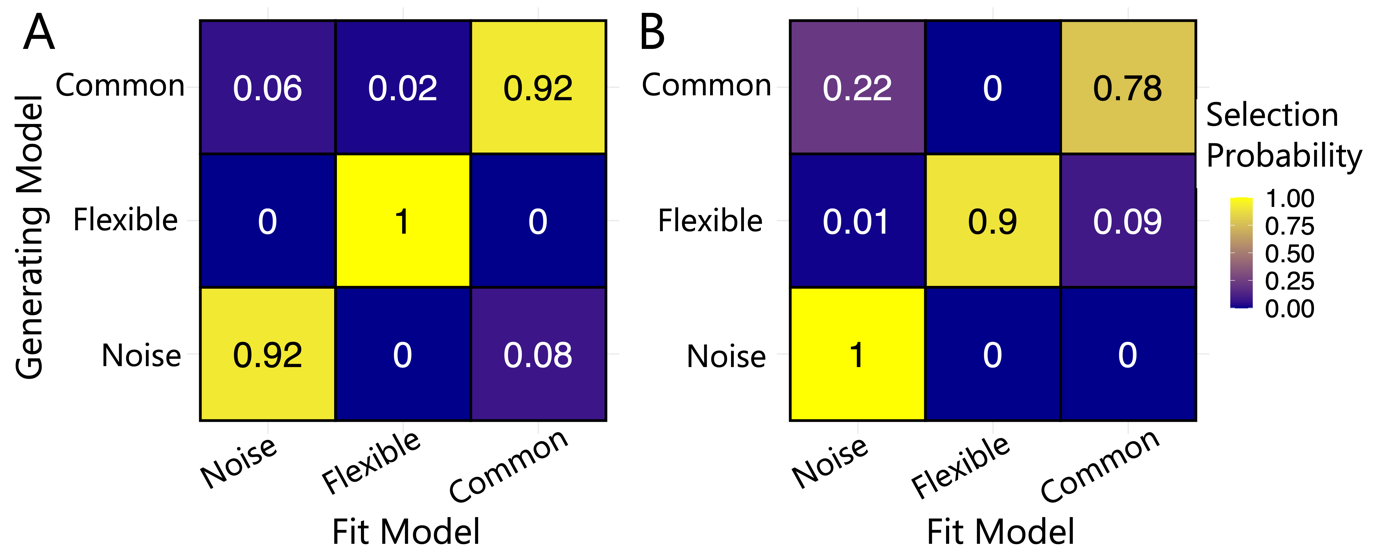


**Fig B. Model Recovery for Models Used To Compare Parameter Settings Across Modalities.** Numbers and colours denote the probability that the data generated with model X are best fit by model Y. (A) Selection probabilities calculated using AIC for model selection. (B) Selection probabilities calculated using BIC for model selection.
