# Supplementary material for: Modality independent or modality specific? Common computations underlie confidence judgements in visual and auditory decisions
Source: PLoS Comput Biol. 2023 Jul 14;19(7):e1011245. doi: 10.1371/journal.pcbi.1011245 (PMC10426961; doi:10.1371/journal.pcbi.1011245)
Supplement: S5 Text — Fig A. Parameter Recovery for Distance Model. Table A. Correlations Between Generating and Recovered Parameters for Unscaled Evidence Strength Models. Fig B. Parameter Recovery for Standard Bayesian Model. Fig C. Parameter Recovery for Bayesian Model with Free Category Distribution Parameters. Fig D. Parameter Recovery for Bayesian Model with Orientation Dependent Noise. Fig E. Parameter Recovery for Bayesian Model with Decision Noise. Table B: Correlations Between Generating and Recovered Parameters for Bayesian Models. Fig F. Parameter Recovery for Linear Model. Fig G. Parameter Recovery for Quadratic Model. Fig H. Parameter Recovery for Free-Exponent Model. Fig I. Parameter Recovery for Free-Exponent Model with Orientation Dependent Noise. Table C. Correlations Between Generating and Recovered Parameters for Scaled Evidence Strength Models. Fig J. Parameter Recovery with Evenly Spaced Stimulus Values. Table D. Correlations Between Generating and Recovered Parameters for Models with Evenly Spaced Stimulus Values (DOCX) [file pcbi.1011245.s005.docx]

**S5 Text: Parameter Recovery for Different SDs Task Models**

We also explored the parameter recovery properties of the core models: distance, linear, quadratic, free-exponent, free-exponent with orientation dependent noise, log posterior probability ratio, log posterior probability ratio with free category distribution parameters, the log posterior probability ratio with decision noise and the log posterior probability ratio with orientation dependent noise. Data were simulated using a set of parameters for a given model. We then fit the simulated data with the same model and assessed whether we were able to recover the data-generating parameter values from the fitting procedure. We used stimuli that were similar to those used in the different SDs task, resampling stimulus values from the category distributions. To examine the effect of sample size on parameter recovery, we simulated data sets with sample sizes of: a) 360 trials (half the number of trials per participant in the main experiment), b) 720 trials (the same number of trials per participant), c) 1440 trials and d) 7200 trials. As seen in **Fig A** - **Fig I** and **Table A** - **Table C**, as the sample size increased, the parameters were recovered better, as expected. However, even at smaller sample sizes, the parameters were recovered quite well for all models. Of note, some of the more extreme boundary parameters (boundary 6 and boundary 7) did not recover as well, especially at low sample sizes. This was particularly noticeable for the Bayesian models (**Fig B - Fig E**). We suspected that this was because, by definition, the majority of stimulus values were sampled from around the means of the category distributions and thus, there were fewer simulated responses to constrain estimates of the boundaries further away from the means. We investigated this by sampling stimulus values that were equally spaced between a reasonable minimum and maximum value of size 720 trials. We simulated responses according to the fixed, free-exponent and Bayesian models and then determined whether the recovery of the generating parameters had improved. As shown in **Fig J** and **Table D**, the same parameters, particularly boundary 6 and boundary 7, were recovered better with the evenly spaced data.


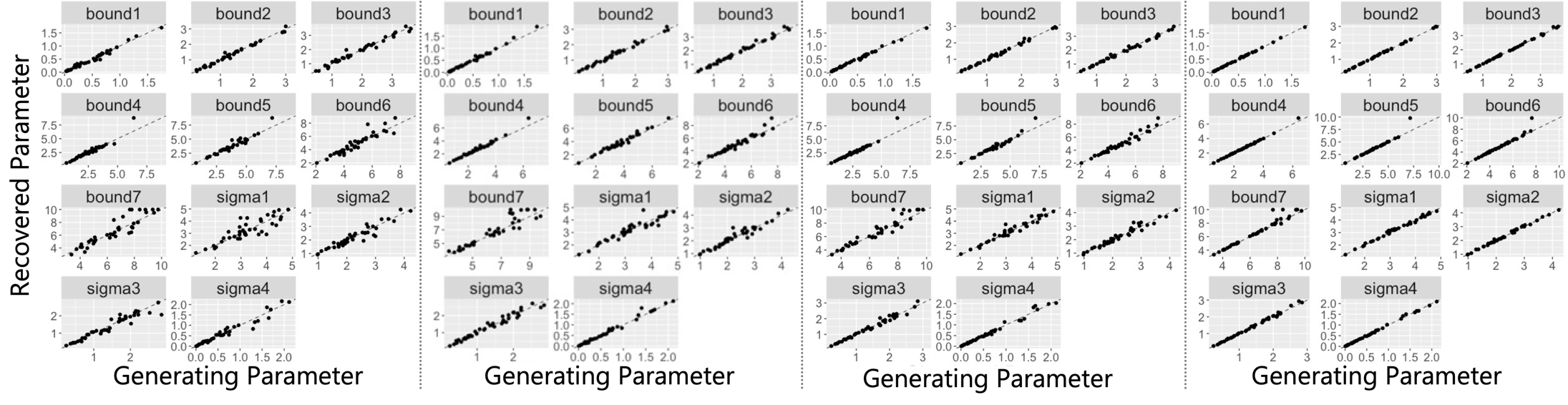


**Fig A. Parameter Recovery for Distance Model.** We randomly generated stimulus values and simulated category and confidence responses using a set of parameters (generating parameters plotted on the x axis). We then fit the model to these simulated responses to determine whether we could recover the generating parameters (recovered parameters plotted on the y axis). The simulated datasets consisted of either 360 (first panel from left), 720 (second panel), 1440 (third panel) and 7200 trials (fourth panel). Bound 1 – 7 refer to the category/confidence boundaries and sigma 1 – 4 refer to the estimated noise parameters at each intensity level (the perceived level of sensory uncertainty in the observer’s perception of the stimulus). Dotted diagonal lines indicate perfect recovery of the data-generating parameter values.

**Table A**

***Correlations Between Generating and Recovered Parameters for Unscaled Evidence Strength Models***

| Parameter | Size of Simulated Dataset (Trials) | | | |
| --- | --- | --- | --- | --- |
|  | 360 | 720 | 1440 | 7200 |
| Distance Model | | | | |
| Bound 1 | 0.99 | 1.00 | 1.00 | 1.00 |
| Bound 2 | 0.99 | 0.99 | 1.00 | 1.00 |
| Bound 3 | 0.99 | 0.99 | 1.00 | 1.00 |
| Bound 4 | 0.96 | 0.99 | 0.96 | 1.00 |
| Bound 5 | 0.96 | 0.99 | 0.98 | 0.97 |
| Bound 6 | 0.94 | 0.97 | 0.98 | 0.98 |
| Bound 7 | 0.92 | 0.93 | 0.95 | 0.98 |
| Sigma 1 | 0.87 | 0.95 | 0.97 | 1.00 |
| Sigma 2 | 0.95 | 0.96 | 0.98 | 1.00 |
| Sigma 3 | 0.96 | 0.98 | 0.99 | 1.00 |
| Sigma 4 | 0.98 | 0.99 | 0.99 | 1.00 |

**
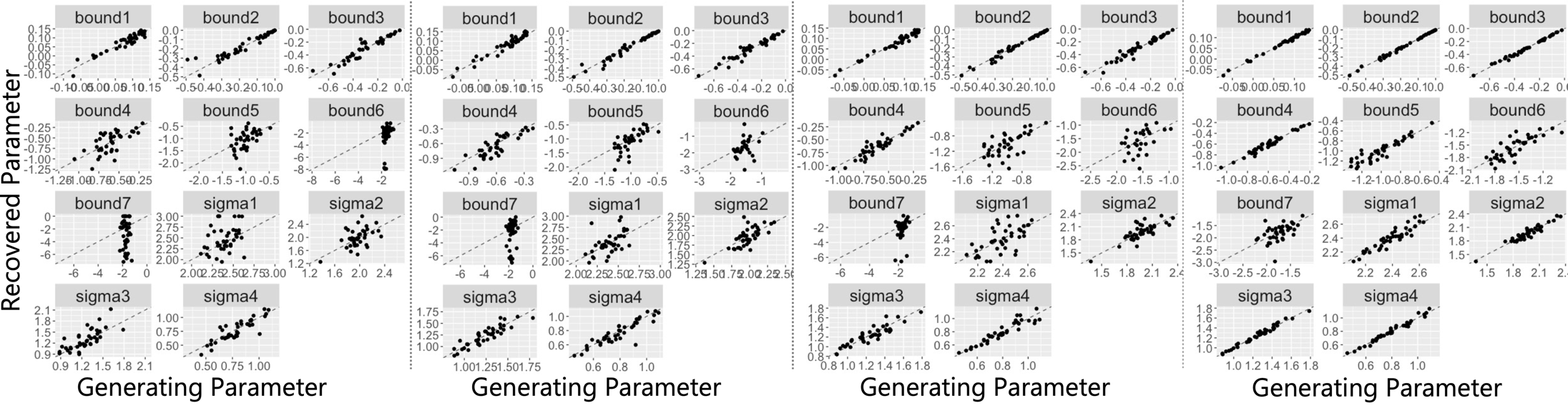
**

**Fig B. Parameter Recovery for Standard Bayesian Model.** We randomly generated stimulus values and simulated category and confidence responses using a set of parameters (generating parameters plotted on the x axis) according to the standard Bayesian model (log posterior probability ratio model). We then fit the model to these simulated responses to determine whether we could recover the generating parameters (recovered parameters plotted on the y axis). The simulated datasets consisted of either 360 (first panel from left), 720 (second panel), 1440 (third panel) and 7200 trials (fourth panel). Bound 1 – 7 refer to the category/confidence boundaries in *d* units (log posterior probability ratio space) and sigma 1 – 4 refer to the estimated noise parameters at each intensity level (the perceived level of sensory uncertainty in the observer’s perception of the stimulus). Dotted diagonal lines indicate perfect recovery of the data-generating parameter values.

**
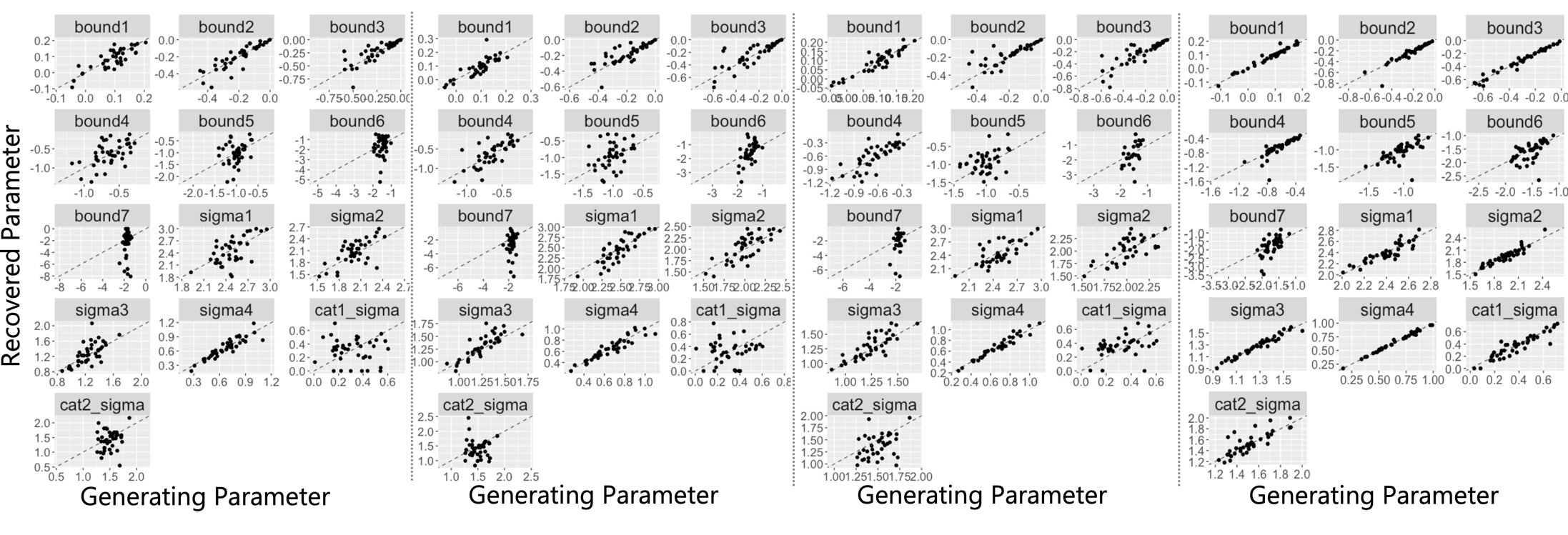
**

**Fig C. Parameter Recovery for Bayesian Model with Free Category Distribution Parameters.** We randomly generated stimulus values and simulated category and confidence responses using a set of parameters (generating parameters plotted on the x axis) according to the Bayesian model with free category distribution parameters. We then fit the model to these simulated responses to determine whether we could recover the generating parameters (recovered parameters plotted on the y axis). The simulated datasets consisted of either 360 (first panel from left), 720 (second panel), 1440 (third panel) and 7200 trials (fourth panel). Bound 1 – 7 refer to the category/confidence boundaries in *d* units (log posterior probability ratio space), sigma 1 – 4 refer to the estimated noise parameters at each intensity level (the perceived level of sensory uncertainty in the observer’s perception of the stimulus), ‘cat1_sigma’ refers to the estimated standard deviation of the category 1 distribution and ‘cat2_sigma’ refers to the estimated standard deviation of the category 2 distribution. Dotted diagonal lines indicate perfect recovery of the data-generating parameter values.


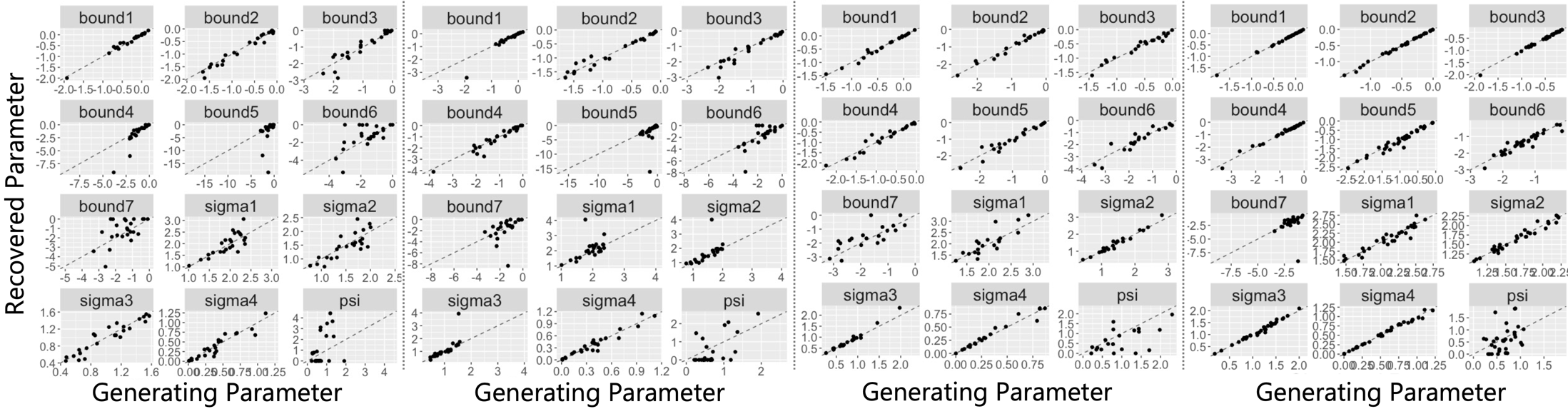


**Fig D. Parameter Recovery for Bayesian Model with Orientation Dependent Noise.** We randomly generated stimulus values and simulated category and confidence responses using a set of parameters (generating parameters plotted on the x axis) according to the Bayesian model with orientation dependent noise. We then fit the model to these simulated responses to determine whether we could recover the generating parameters (recovered parameters plotted on the y axis). The simulated datasets consisted of either 360 (first panel from left), 720 (second panel), 1440 (third panel) and 7200 trials (fourth panel). Bound 1 – 7 refer to the category/confidence boundaries in *d* units (log posterior probability ratio space), sigma 1 – 4 refer to the estimated noise parameters at each intensity level (the perceived level of sensory uncertainty in the observer’s perception of the stimulus) and ‘psi’ refers to the orientation dependent noise parameter (see Equation 2). Dotted diagonal lines indicate perfect recovery of the data-generating parameter values.

**
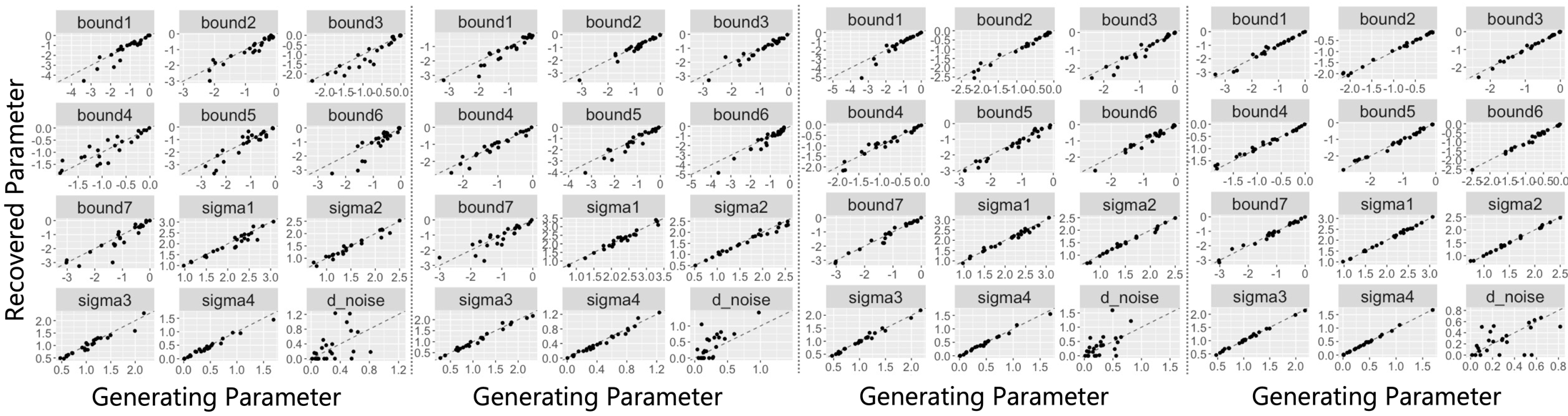
**

**Fig E. Parameter Recovery for Bayesian Model with Decision Noise.** We randomly generated stimulus values and simulated category and confidence responses using a set of parameters (generating parameters plotted on the x axis) according to the Bayesian model with decision noise. We then fit the model to these simulated responses to determine whether we could recover the generating parameters (recovered parameters plotted on the y axis). The simulated datasets consisted of either 360 (first panel from left), 720 (second panel), 1440 (third panel) and 7200 trials (fourth panel). Bound 1 – 7 refer to the category/confidence boundaries in *d* units (log posterior probability ratio space), sigma 1 – 4 refer to the estimated noise parameters at each intensity level (the perceived level of sensory uncertainty in the observer’s perception of the stimulus), and ‘d_noise’ refers to the decision noise parameter (a Gaussian noise term on *d*). Dotted diagonal lines indicate perfect recovery of the data-generating parameter values.

**Table B**

***Correlations Between Generating and Recovered Parameters for Bayesian Models***

|  | Parameter | Size of Simulated Dataset (Trials) | | | |
| --- | --- | --- | --- | --- | --- |
|  |  | 360 | 720 | 1440 | 7200 |
|  |  | Standard Bayesian Model | | | |
|  | Bound 1 | 0.95 | 0.96 | 0.98 | 1.00 |
|  | Bound 2 | 0.95 | 0.98 | 0.99 | 1.00 |
|  | Bound 3 | 0.94 | 0.96 | 0.97 | 1.00 |
|  | Bound 4 | 0.67 | 0.85 | 0.93 | 0.98 |
|  | Bound 5 | 0.50 | 0.71 | 0.62 | 0.92 |
|  | Bound 6 | 0.38 | 0.31 | 0.46 | 0.81 |
|  | Bound 7 | -0.02 | 0.22 | 0.29 | 0.51 |
|  | Sigma 1 | 0.34 | 0.54 | 0.62 | 0.93 |
|  | Sigma 2 | 0.50 | 0.72 | 0.78 | 0.95 |
|  | Sigma 3 | 0.73 | 0.90 | 0.91 | 0.99 |
|  | Sigma 4 | 0.87 | 0.91 | 0.94 | 0.99 |
|  |  | Bayesian Model with Free Category Distribution Parameters | | | |
|  | Bound 1 | 0.86 | 0.85 | 0.92 | 0.98 |
|  | Bound 2 | 0.90 | 0.91 | 0.81 | 0.96 |
|  | Bound 3 | 0.87 | 0.91 | 0.84 | 0.97 |
|  | Bound 4 | 0.67 | 0.70 | 0.77 | 0.88 |
|  | Bound 5 | 0.17 | 0.36 | 0.34 | 0.71 |
|  | Bound 6 | 0.10 | 0.34 | 0.49 | 0.74 |
|  | Bound 7 | 0.04 | 0.03 | 0.34 | 0.59 |
|  | Cat. 1 Sigma | 0.24 | 0.50 | 0.39 | 0.17 |
|  | Cat. 2 Sigma | 0.70 | 0.67 | 0.34 | 0.58 |
|  | Sigma 1 | 0.65 | 0.46 | 0.69 | 0.93 |
|  | Sigma 2 | 0.60 | 0.64 | 0.68 | 0.88 |
|  | Sigma 3 | 0.51 | 0.76 | 0.82 | 0.90 |
|  | Sigma 4 | 0.90 | 0.95 | 0.97 | 0.98 |
|  |  | Bayesian Model with Orientation Dependent Noise | | | |
|  | Bound 1 | 0.99 | 0.96 | 0.99 | 1.00 |
|  | Bound 2 | 0.98 | 0.98 | 0.99 | 1.00 |
|  | Bound 3 | 0.94 | 0.96 | 0.98 | 1.00 |
|  | Bound 4 | 0.89 | 0.96 | 0.97 | 0.99 |
|  | Bound 5 | 0.35 | 0.29 | 0.96 | 0.97 |
|  | Bound 6 | 0.61 | 0.73 | 0.94 | 0.94 |
|  | Bound 7 | 0.56 | 0.40 | 0.77 | 0.15 |
|  | Psi | 0.43 | 0.60 | 0.57 | 0.40 |
|  | Sigma 1 | 0.80 | 0.53 | 0.85 | 0.95 |
|  | Sigma 2 | 0.81 | 0.64 | 0.98 | 0.98 |
|  | Sigma 3 | 0.94 | 0.73 | 0.99 | 0.99 |
|  | Sigma 4 | 0.93 | 0.96 | 0.98 | 0.99 |

|  |  | Bayesian Model with Decision Noise | | | |
| --- | --- | --- | --- | --- | --- |
|  | Bound 1 | 0.93 | 0.95 | 0.96 | 0.99 |
|  | Bound 2 | 0.95 | 0.97 | 0.99 | 1.00 |
|  | Bound 3 | 0.95 | 0.96 | 0.97 | 1.00 |
|  | Bound 4 | 0.89 | 0.96 | 0.97 | 0.99 |
|  | Bound 5 | 0.92 | 0.96 | 0.98 | 0.99 |
|  | Bound 6 | 0.89 | 0.97 | 0.97 | 0.99 |
|  | Bound 7 | 0.92 | 0.87 | 0.98 | 0.99 |
|  | Dnoise | 0.45 | 0.72 | 0.60 | 0.54 |
|  | Sigma 1 | 0.96 | 0.98 | 0.99 | 1.00 |
|  | Sigma 2 | 0.97 | 0.99 | 0.99 | 1.00 |
|  | Sigma 3 | 0.97 | 0.99 | 0.99 | 1.00 |
|  | Sigma 4 | 0.99 | 0.99 | 0.99 | 1.00 |

**
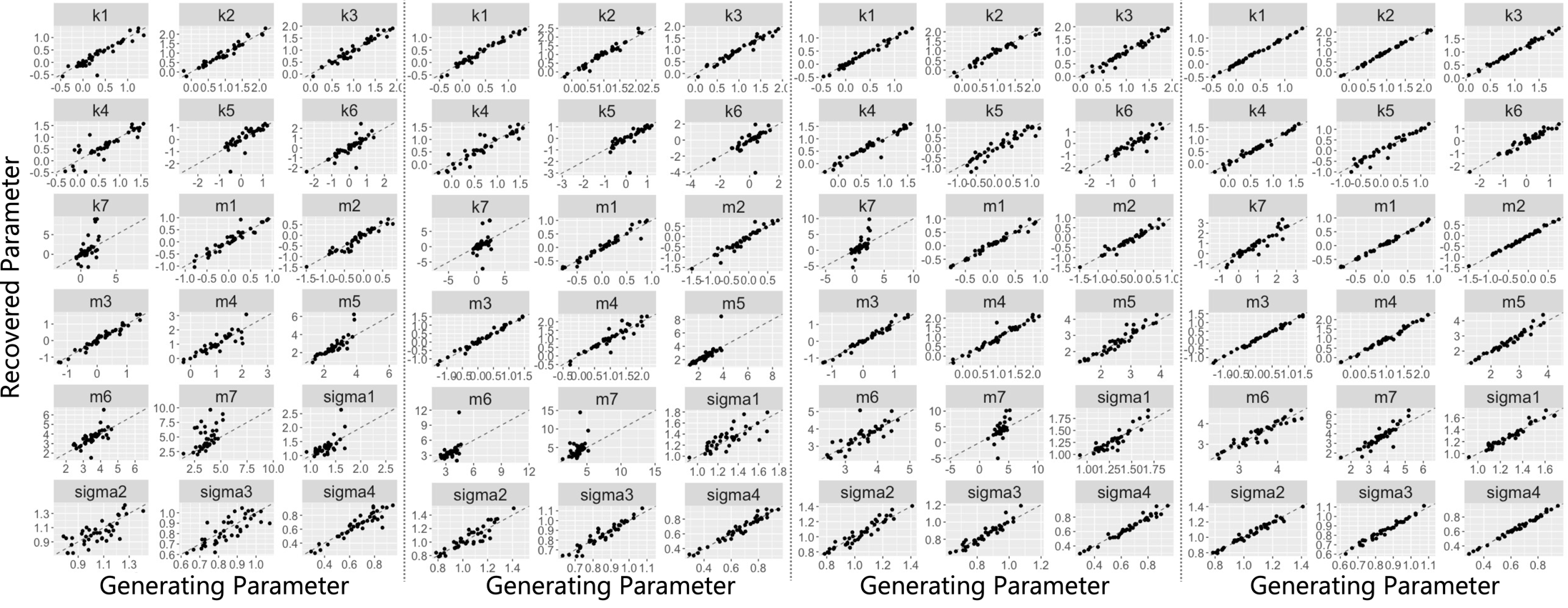
**

**Fig F. Parameter Recovery for Linear Model.** We randomly generated stimulus values and simulated category and confidence responses using a set of parameters (generating parameters plotted on the x axis) according to the linear model. We then fit the model to these simulated responses to determine whether we could recover the generating parameters (recovered parameters plotted on the y axis). The simulated datasets consisted of either 360 (first panel from left), 720 (second panel), 1440 (third panel) and 7200 trials (fourth panel). Sigma 1 – 4 refer to the estimated noise parameters at each intensity level (the perceived level of sensory uncertainty in the observer’s perception of the stimulus). Parameters *k* 1 – 7 and *m* 1 – 7 are used to estimate the category/confidence boundaries according to $b=k+m\sigma$ (see description for scaled evidence strength models for more details). Dotted diagonal lines indicate perfect recovery of the data-generating parameter values.

**
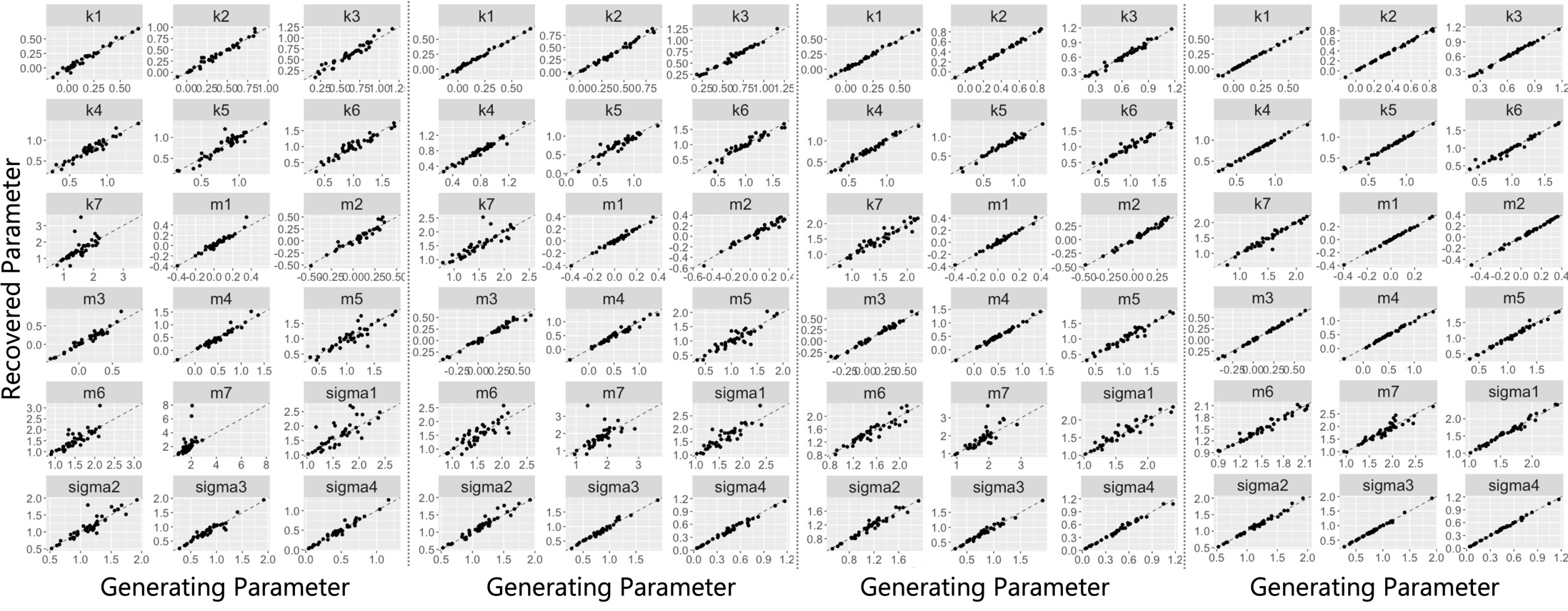
**

**Fig G. Parameter Recovery for Quadratic Model.** We randomly generated stimulus values and simulated category and confidence responses using a set of parameters (generating parameters plotted on the x axis) according to the quadratic model. We then fit the model to these simulated responses to determine whether we could recover the generating parameters (recovered parameters plotted on the y axis). The simulated datasets consisted of either 360 (first panel from left), 720 (second panel), 1440 (third panel) and 7200 trials (fourth panel). Sigma 1 – 4 refer to the estimated noise parameters at each intensity level (the perceived level of sensory uncertainty in the observer’s perception of the stimulus). Parameters *k* 1 – 7 and *m* 1 – 7 are used to estimate the category/confidence boundaries according to $b=k+m\sigma^{2}$ (see description for scaled evidence strength models). Dotted diagonal lines indicate perfect recovery of the data-generating parameter values.

**
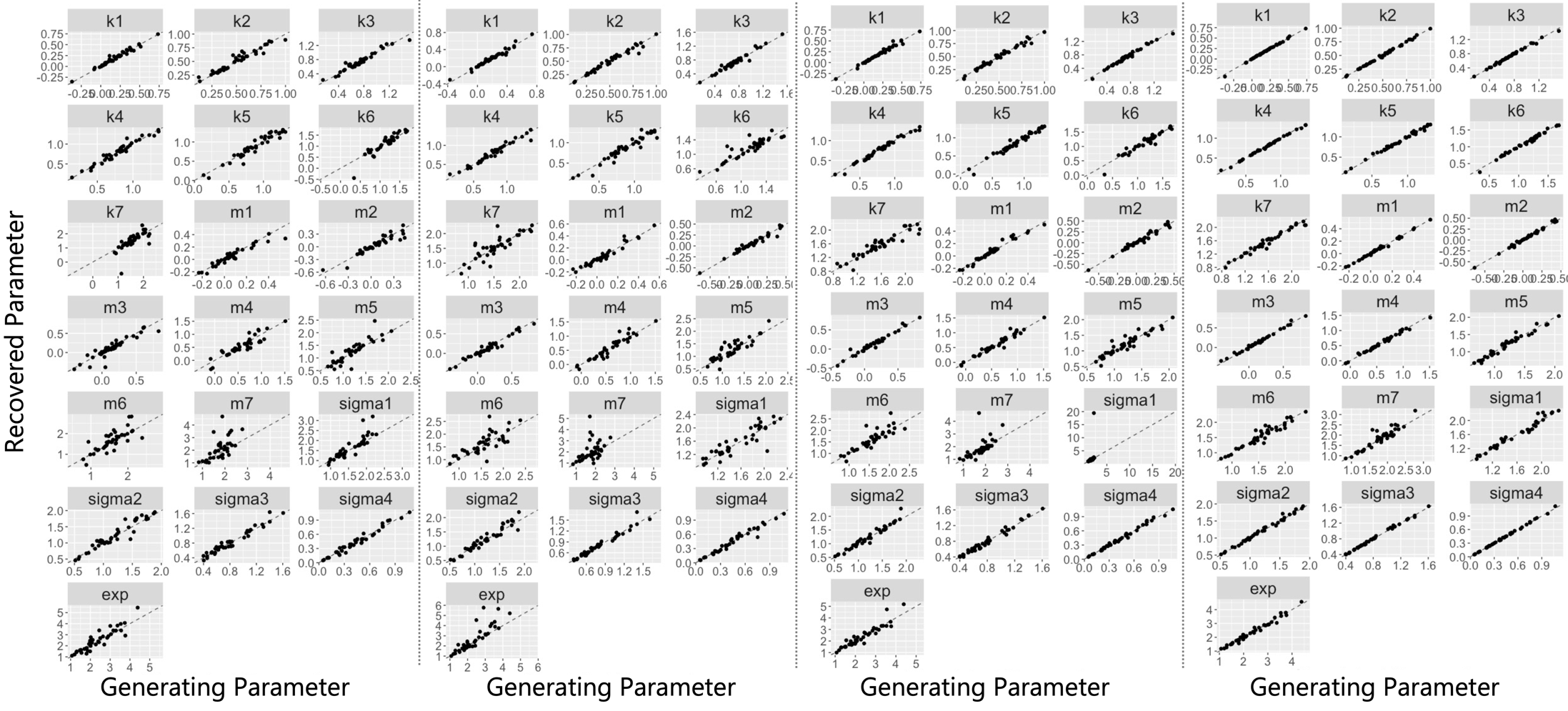
**

**Fig H. Parameter Recovery for Free-Exponent Model.** We randomly generated stimulus values and simulated category and confidence responses using a set of parameters (generating parameters plotted on the x axis) according to the free-exponent model. We then fit the model to these simulated responses to determine whether we could recover the generating parameters (recovered parameters plotted on the y axis). The simulated datasets consisted of either 360 (first panel from left), 720 (second panel), 1440 (third panel) and 7200 trials (fourth panel). Sigma 1 – 4 refer to the estimated noise parameters at each intensity level (the perceived level of sensory uncertainty in the observer’s perception of the stimulus). Parameters ‘exp’ (the exponent), *k* 1 – 7 and *m* 1 – 7 are used to estimate the category/confidence boundaries according to $b=k+m\sigma^{exp}$ (see description for scaled evidence strength models). Dotted diagonal lines indicate perfect recovery of the data-generating parameter values.

**
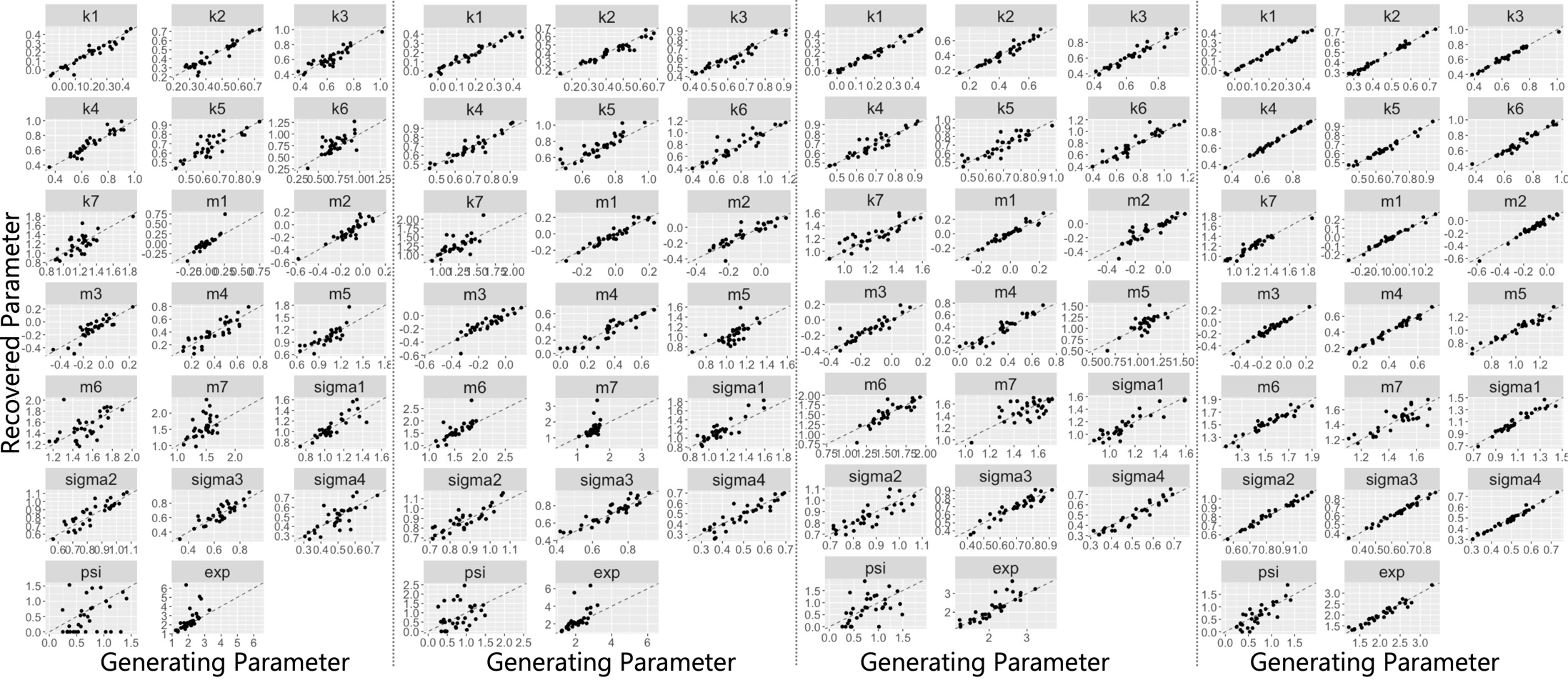
**

**Fig I. Parameter Recovery for Free-Exponent Model with Orientation Dependent Noise.** We randomly generated stimulus values and simulated category and confidence responses using a set of parameters (generating parameters plotted on the x axis) according to the free-exponent model with orientation dependent noise. We then fit the model to these simulated responses to determine whether we could recover the generating parameters (recovered parameters plotted on the y axis). The simulated datasets consisted of either 360 (first panel from left), 720 (second panel), 1440 (third panel) and 7200 trials (fourth panel). Sigma 1 – 4 refer to the estimated noise parameters at each intensity level (the perceived level of sensory uncertainty in the observer’s perception of the stimulus) and ‘psi’ refers to the orientation dependent noise parameter (see Equation 2). Parameters ‘exp’ (the exponent), *k* 1 – 7 and *m* 1 – 7 are used to estimate the category/confidence boundaries according to xf$b=k+m\sigma^{exp}$ (see description for scaled evidence strength models). Dotted diagonal lines indicate perfect recovery of the data-generating parameter values.

**Table C**

***Correlations Between Generating and Recovered Parameters for Scaled Evidence Strength Models***

| Parameter | Size of Simulated Dataset | | | |
| --- | --- | --- | --- | --- |
|  | 360 | 720 | 1440 | 7200 |
| Linear Model | | | | |
| k1 | 0.94 | 0.98 | 0.99 | 1.00 |
| k2 | 0.97 | 0.98 | 0.98 | 1.00 |
| k3 | 0.97 | 0.98 | 0.97 | 0.99 |
| k4 | 0.86 | 0.92 | 0.97 | 0.99 |
| k5 | 0.80 | 0.71 | 0.94 | 0.97 |
| k6 | 0.82 | 0.66 | 0.91 | 0.97 |
| k7 | 0.61 | 0.46 | 0.71 | 0.94 |
| m1 | 0.95 | 0.97 | 0.99 | 1.00 |
| m2 | 0.95 | 0.96 | 0.97 | 1.00 |
| m3 | 0.98 | 0.99 | 0.99 | 1.00 |
| m4 | 0.87 | 0.93 | 0.97 | 0.99 |
| m5 | 0.86 | 0.75 | 0.95 | 0.98 |
| m6 | 0.69 | 0.49 | 0.82 | 0.93 |
| m7 | 0.51 | 0.43 | 0.62 | 0.90 |
| Sigma 1 | 0.65 | 0.77 | 0.91 | 0.97 |
| Sigma 2 | 0.76 | 0.90 | 0.95 | 0.98 |
| Sigma 3 | 0.79 | 0.95 | 0.95 | 0.99 |
| Sigma 4 | 0.93 | 0.97 | 0.98 | 1.00 |

| Quadratic Model | | | | |
| --- | --- | --- | --- | --- |
| k1 | 0.99 | 1.00 | 1.00 | 1.00 |
| k2 | 0.98 | 0.99 | 1.00 | 1.00 |
| k3 | 0.96 | 0.99 | 0.99 | 1.00 |
| k4 | 0.96 | 0.98 | 0.99 | 1.00 |
| k5 | 0.94 | 0.94 | 0.98 | 0.99 |
| k6 | 0.96 | 0.96 | 0.97 | 0.98 |
| k7 | 0.69 | 0.86 | 0.95 | 0.97 |
| m1 | 0.97 | 0.99 | 0.99 | 1.00 |
| m2 | 0.95 | 0.98 | 0.99 | 1.00 |
| m3 | 0.96 | 0.99 | 0.99 | 1.00 |
| m4 | 0.97 | 0.98 | 0.99 | 1.00 |
| m5 | 0.89 | 0.91 | 0.97 | 0.99 |
| m6 | 0.80 | 0.82 | 0.92 | 0.98 |
| m7 | 0.45 | 0.57 | 0.78 | 0.94 |
| Sigma 1 | 0.77 | 0.88 | 0.94 | 0.99 |
| Sigma 2 | 0.90 | 0.96 | 0.97 | 0.99 |
| Sigma 3 | 0.96 | 0.99 | 0.98 | 1.00 |
| Sigma 4 | 0.98 | 0.99 | 0.99 | 1.00 |
| Free-Exponent Model | | | | |
| Exponent | 0.92 | 0.87 | 0.93 | 0.99 |
| k1 | 0.98 | 0.98 | 0.99 | 1.00 |
| k2 | 0.97 | 0.98 | 0.99 | 1.00 |
| k3 | 0.97 | 0.98 | 0.99 | 1.00 |
| k4 | 0.97 | 0.98 | 0.95 | 1.00 |
| k5 | 0.96 | 0.96 | 0.91 | 1.00 |
| k6 | 0.93 | 0.90 | 0.95 | 0.99 |
| k7 | 0.71 | 0.84 | 0.89 | 0.98 |
| m1 | 0.95 | 0.97 | 0.99 | 1.00 |
| m2 | 0.94 | 0.98 | 0.98 | 1.00 |
| m3 | 0.92 | 0.98 | 0.97 | 0.99 |
| m4 | 0.91 | 0.95 | 0.97 | 0.99 |
| m5 | 0.88 | 0.88 | 0.96 | 0.98 |
| m6 | 0.78 | 0.76 | 0.91 | 0.96 |
| m7 | 0.63 | 0.44 | 0.78 | 0.93 |
| Sigma 1 | 0.88 | 0.89 | 0.94 | 0.98 |
| Sigma 2 | 0.96 | 0.96 | 0.95 | 0.99 |
| Sigma 3 | 0.97 | 0.98 | 0.99 | 1.00 |
| Sigma 4 | 0.98 | 0.98 | 0.99 | 1.00 |

| Free-Exponent Model with Orientation Dependent Noise | | | | |
| --- | --- | --- | --- | --- |
| Exponent | 0.57 | 0.64 | 0.87 | 0.97 |
| k1 | 0.95 | 0.98 | 0.99 | 1.00 |
| k2 | 0.94 | 0.95 | 0.96 | 0.99 |
| k3 | 0.90 | 0.95 | 0.97 | 0.99 |
| k4 | 0.94 | 0.93 | 0.93 | 1.00 |
| k5 | 0.84 | 0.86 | 0.88 | 0.99 |
| k6 | 0.69 | 0.90 | 0.93 | 0.98 |
| k7 | 0.73 | 0.69 | 0.84 | 0.96 |
| m1 | 0.88 | 0.95 | 0.96 | 0.99 |
| m2 | 0.89 | 0.89 | 0.89 | 0.98 |
| m3 | 0.88 | 0.88 | 0.91 | 0.98 |
| m4 | 0.86 | 0.92 | 0.95 | 0.99 |
| m5 | 0.76 | 0.66 | 0.77 | 0.97 |
| m6 | 0.58 | 0.74 | 0.87 | 0.95 |
| m7 | 0.48 | 0.50 | 0.62 | 0.81 |
| Psi | 0.34 | 0.32 | 0.47 | 0.83 |
| Sigma 1 | 0.85 | 0.88 | 0.90 | 0.96 |
| Sigma 2 | 0.87 | 0.85 | 0.82 | 0.99 |
| Sigma 3 | 0.86 | 0.92 | 0.93 | 0.98 |
| Sigma 4 | 0.77 | 0.90 | 0.95 | 0.99 |

**
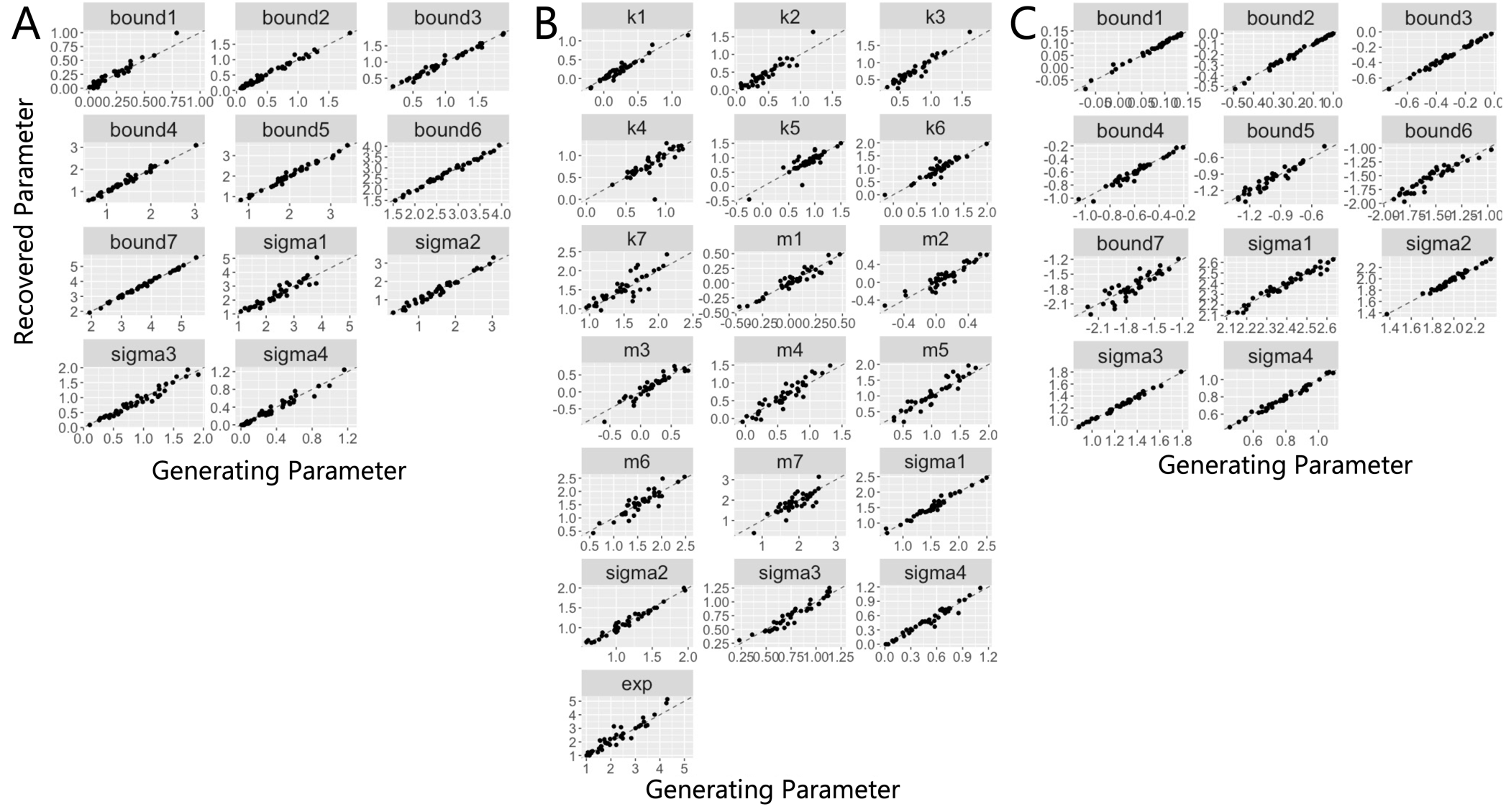
**

**Fig J. Parameter Recovery with Evenly Spaced Stimulus Values.** To determine how the parameter recoveries were influenced by the sampling of stimulus values, rather than sample values from the category distributions we used values that were evenly spaced between a reasonable minimum and maximum. We simulated category and confidence responses according to (A) the fixed, (B) the free-exponent and (C) the standard Bayesian model. We used 720 trials per simulated dataset and found that the same parameters, particularly boundary 6 and boundary 7, were recovered better with the evenly spaced data.

**Table D**

***Correlations Between Generating and Recovered Parameters for Models with Evenly Spaced Stimulus Values***

| Distance Model | | Free Exponent Model | | Standard Bayesian Model | |
| --- | --- | --- | --- | --- | --- |
| Parameter | Correlation | Parameter | Correlation | Parameter | Correlation |
| Bound 1 | 0.97 | Exponent | 0.95 | Bound 1 | 0.99 |
| Bound 2 | 0.99 | k1 | 0.96 | Bound 2 | 1.00 |
| Bound 3 | 0.99 | k2 | 0.92 | Bound 3 | 1.00 |
| Bound 4 | 0.99 | k3 | 0.96 | Bound 4 | 0.98 |
| Bound 5 | 0.99 | k4 | 0.80 | Bound 5 | 0.96 |
| Bound 6 | 0.99 | k5 | 0.86 | Bound 6 | 0.95 |
| Bound 7 | 1.00 | k6 | 0.91 | Bound 7 | 0.89 |
| Sigma 1 | 0.93 | k7 | 0.86 | Sigma 1 | 0.98 |
| Sigma 2 | 0.98 | m1 | 0.95 | Sigma 2 | 0.99 |
| Sigma 3 | 0.98 | m2 | 0.93 | Sigma 3 | 1.00 |
| Sigma 4 | 0.98 | m3 | 0.94 | Sigma 4 | 0.99 |
|  |  | m4 | 0.93 |  |  |
|  |  | m5 | 0.94 |  |  |
|  |  | m6 | 0.91 |  |  |
|  |  | m7 | 0.80 |  |  |
|  |  | Sigma 1 | 0.98 |  |  |
|  |  | Sigma 2 | 0.98 |  |  |
|  |  | Sigma 3 | 0.96 |  |  |
|  |  | Sigma 4 | 0.98 |  |  |
