# Supplementary material for: Modality independent or modality specific? Common computations underlie confidence judgements in visual and auditory decisions
Source: PLoS Comput Biol. 2023 Jul 14;19(7):e1011245. doi: 10.1371/journal.pcbi.1011245 (PMC10426961; doi:10.1371/journal.pcbi.1011245)
Supplement: S6 Text — Fig A. Parameter Recovery for Common Settings Model. Fig B. Parameter Recovery for Different Noise Settings Model. Fig C. Parameter Recovery for Flexible Settings Model. Table A. Correlations Between Generating and Recovered Parameters for Cross-Modal Models (DOCX) [file pcbi.1011245.s006.docx]

**S6 Text: Parameter Recovery for Cross-Modal Models**

We also performed parameter recoveries for the models used to explore the parameter settings of the free-exponent model across modalities (see **Fig 7**). We used the same procedure as with the core models, but we simulated data sets with a sample size of 720 trials. **Fig A - Fig C** show parameter recoveries for the common settings, different noise settings and flexible settings models. Correlations for simulated and fitted parameters are reported in **Table A**. The parameters recovered well for all models.

**
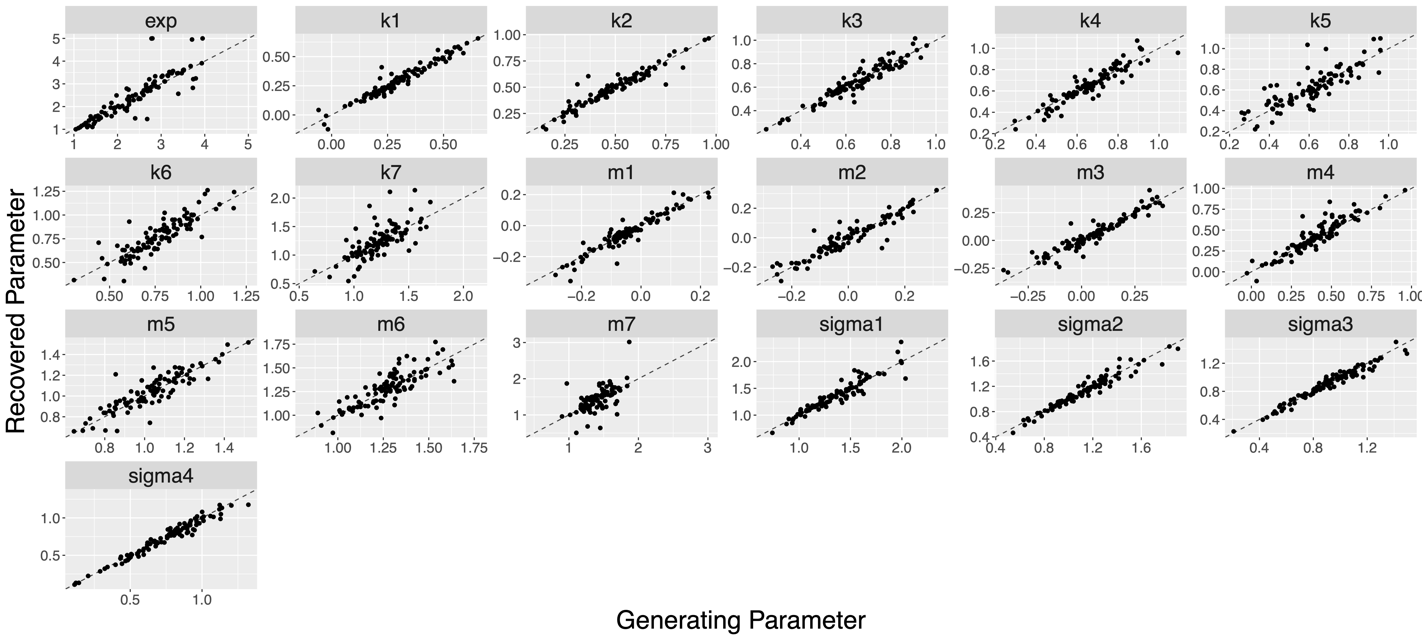
**

**Fig A. Parameter Recovery for Common Settings Model.** We randomly generated visual and auditory stimulus values and simulated category and confidence responses using a set of parameters (generating parameters plotted on the x axis) according to the free-exponent common settings model. This model assumed that all parameter values were constant across modalities. We then fit the model to these simulated responses to determine whether we could recover the generating parameters (recovered parameters plotted on the y axis).

**
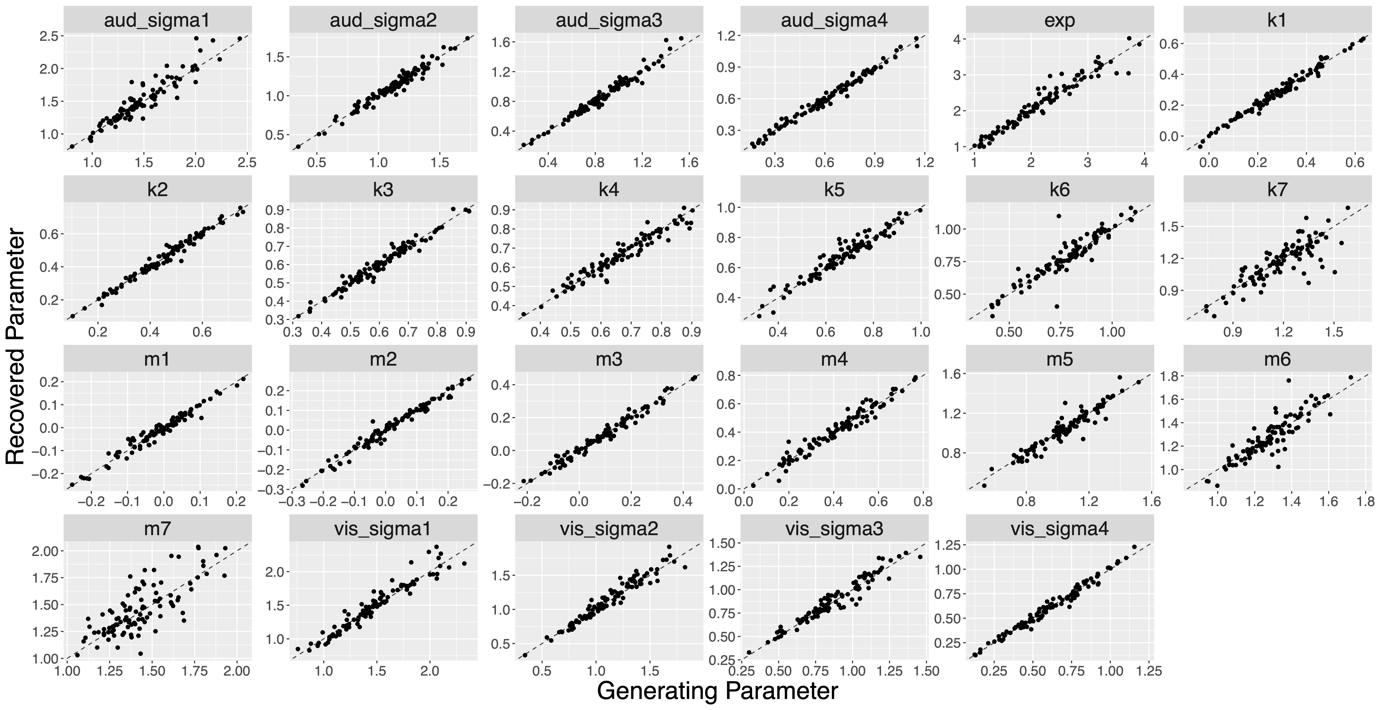
**

**Fig B. Parameter Recovery for Different Noise Settings Model.** We randomly generated visual and auditory stimulus values and simulated category and confidence responses using a set of parameters (generating parameters plotted on the x axis) according to the free-exponent different noise settings model. This model assumed that only the noise (sigma) parameters differed across modalities. We then fit the model to these simulated responses to determine whether we could recover the generating parameters (recovered parameters plotted on the y axis).

**
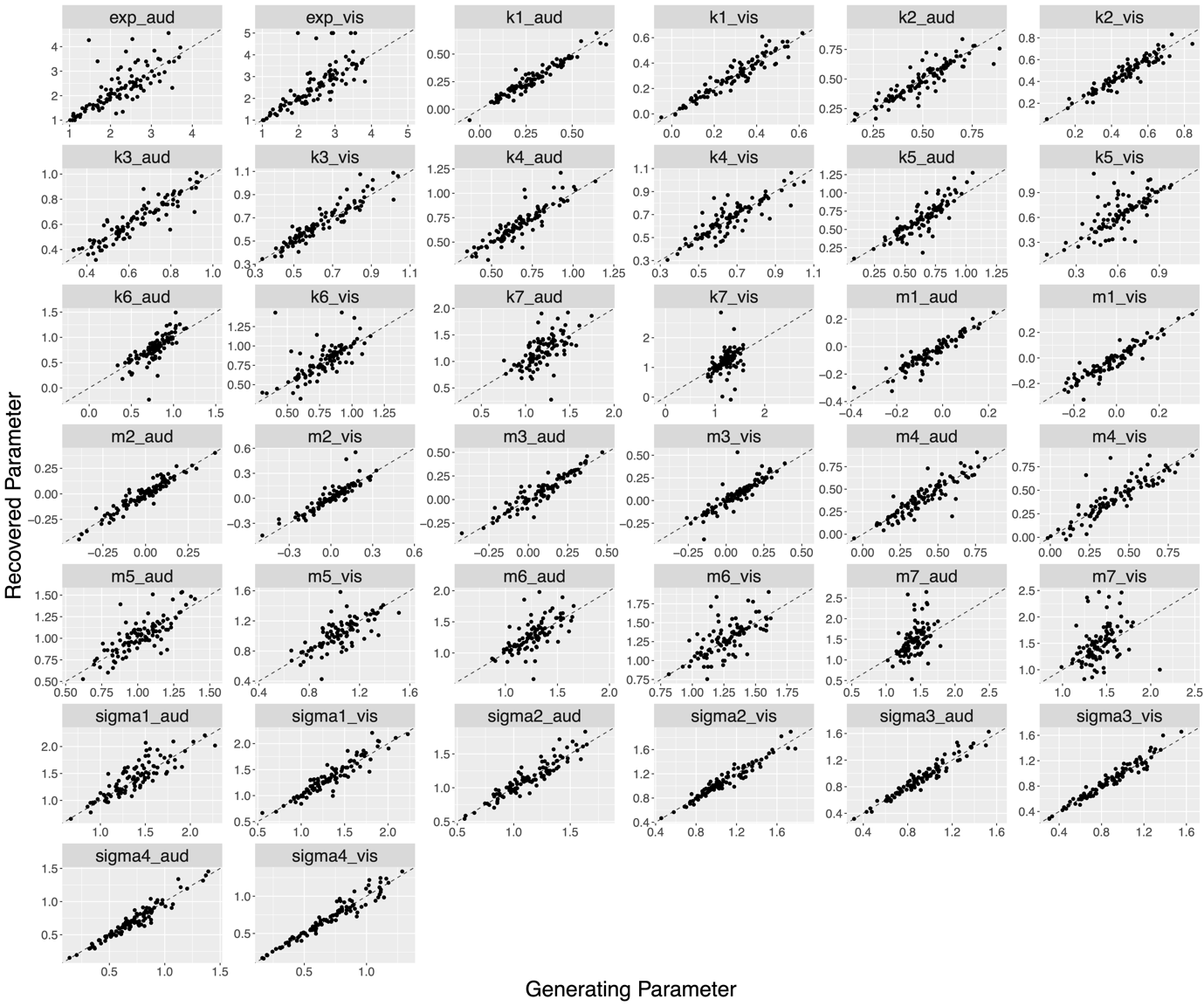
**

**Fig C. Parameter Recovery for Flexible Settings Model.** We randomly generated visual and auditory stimulus values and simulated category and confidence responses using a set of parameters (generating parameters plotted on the x axis) according to the free-exponent flexible settings model. This model assumed that all parameters differed across modalities. We then fit the model to these simulated responses to determine whether we could recover the generating parameters (recovered parameters plotted on the y axis).

**Table A**

***Correlations Between Generating and Recovered Parameters for Cross-Modal Models***

| Common Settings | | Different Noise Settings | | Flexible Settings | |
| --- | --- | --- | --- | --- | --- |
| Parameter | Correlation | Parameter | Correlation | Parameter | Correlation |
| Exponent | 0.88 | Exponent | 0.97 | Exponent Aud | 0.74 |
| k1 | 0.97 | k1 | 0.99 | Exponent Vis | 0.78 |
| k2 | 0.95 | k2 | 0.99 | k1 Aud | 0.96 |
| k3 | 0.95 | k3 | 0.98 | k1 Vis | 0.94 |
| k4 | 0.93 | k4 | 0.96 | k2 Aud | 0.89 |
| k5 | 0.83 | k5 | 0.96 | k2 Vis | 0.94 |
| k6 | 0.87 | k6 | 0.93 | k3 Aud | 0.92 |
| k7 | 0.72 | k7 | 0.83 | k3 Vis | 0.94 |
| m1 | 0.95 | m1 | 0.98 | k4 Aud | 0.9 |
| m2 | 0.94 | m2 | 0.99 | k4 Vis | 0.86 |
| m3 | 0.95 | m3 | 0.99 | k5 Aud | 0.86 |
| m4 | 0.92 | m4 | 0.97 | k5 Vis | 0.72 |
| m5 | 0.88 | m5 | 0.95 | k6 Aud | 0.77 |
| m6 | 0.84 | m6 | 0.89 | k6 Vis | 0.66 |
| m7 | 0.61 | m7 | 0.77 | k7 Aud | 0.6 |
| Sigma 1 | 0.94 | Sigma 1 Aud | 0.94 | k7 Vis | 0.35 |
| Sigma 2 | 0.97 | Sigma 1 Vis | 0.96 | m1 Aud | 0.92 |
| Sigma 3 | 0.98 | Sigma 2 Aud | 0.98 | m1 Vis | 0.94 |
| Sigma 4 | 0.98 | Sigma 2 Vis | 0.97 | m2 Aud | 0.95 |
|  |  | Sigma 3 Aud | 0.98 | m2 Vis | 0.9 |
|  |  | Sigma 3 Vis | 0.97 | m3 Aud | 0.94 |
|  |  | Sigma 4 Aud | 0.99 | m3 Vis | 0.89 |
|  |  | Sigma 4 Vis | 0.99 | m4 Aud | 0.9 |
|  |  |  |  | m4 Vis | 0.89 |
|  |  |  |  | m5 Aud | 0.78 |
|  |  |  |  | m5 Vis | 0.74 |
|  |  |  |  | m6 Aud | 0.68 |
|  |  |  |  | m6 Vis | 0.66 |
|  |  |  |  | m7 Aud | 0.47 |
|  |  |  |  | m7 Vis | 0.36 |
|  |  |  |  | Sigma 1 Aud | 0.85 |
|  |  |  |  | Sigma 1 Vis | 0.94 |
|  |  |  |  | Sigma 2 Aud | 0.93 |
|  |  |  |  | Sigma 2 Vis | 0.97 |
|  |  |  |  | Sigma 3 Aud | 0.97 |
|  |  |  |  | Sigma 3 Vis | 0.97 |
|  |  |  |  | Sigma 4 Aud | 0.96 |
|  |  |  |  | Sigma 4 Vis | 0.97 |
