# Supplementary material for: Modality independent or modality specific? Common computations underlie confidence judgements in visual and auditory decisions
Source: PLoS Comput Biol. 2023 Jul 14;19(7):e1011245. doi: 10.1371/journal.pcbi.1011245 (PMC10426961; doi:10.1371/journal.pcbi.1011245)
Supplement: S7 Text — (DOCX) [file pcbi.1011245.s007.docx]

**S7 Text: Headphone Calibration**

Brüel & Kjær equipment (Brüel & Kjær, Denmark) was used for all auditory calibration. Pure tones were generated and adjusted using Psychophysics Toolbox extensions for MATLAB on a Dell Precision T1700 with an ASIO4ALL sound driver and a sampling rate of 28 kHz to present a flat frequency response between 0.5 and 5 kHz. The flat frequency response was set by coupling the earphone of the Sennheiser HD 202 unit to a Type 5152 artificial ear with a DB0843 adaptor. The artificial ear contained a type 4144 1-inch pressure-field microphone connected to a Type 2250B handheld analyzer (a Class 1 sound level meter under AS/NZS IEC 61672-1 [Standards Australia, 2019]). The analyzer was calibrated yearly by the Brüel & Kjær laboratories in Sydney, Australia, and its status was checked before and after use by coupling it to a Type 4231 sound level calibrator. The adjustments required to present a flat frequency response output between 0.5 and 5 kHz were determined for each earphone of the Sennheiser HD 202 by presenting a 0.5 kHz tone at 85.0 dB SPL, as measured by the system described above. Tones at 0.75 kHz, 1 kHz, 1.5 kHz, 2 kHz, 3 kHz, 4 kHz and 5 kHz were then played in turn and the required adjustments in output level were determined so that each tone presented within the range of 84.0 to 86.0 dB SPL (within 1.0 dB of 85 dB SPL). The consistency of these adjustments were then checked for output levels at half the sound pressure (an output reduction of 6 dB SPL) and one quarter the sound pressure (an output reduction of 12 dB SPL).
