# Supplementary material for: Modality independent or modality specific? Common computations underlie confidence judgements in visual and auditory decisions
Source: PLoS Comput Biol. 2023 Jul 14;19(7):e1011245. doi: 10.1371/journal.pcbi.1011245 (PMC10426961; doi:10.1371/journal.pcbi.1011245)
Supplement: S8 Text — (DOCX) [file pcbi.1011245.s008.docx]

**S8 Text: Derivation of *d* for Different Means and Different SDs Tasks**

Following Adler and Ma (2018), we used **Equation 6** and **Equation 7** for the computation of *d*. We provide the details for derivation of *d* below, in the same way as they are provided in Adler and Ma’s original paper.

The log posterior ratio is equivalent to the log likelihood ratio plus the log prior ratio:

|  | $d=\log\frac{p\left( C=1 \right\vert x)}{p\left( C=2 \right\vert x)}=\log\frac{p\left( x \right\vert C=1)}{p\left( x \right\vert C=2)}+log\frac{p(C=1)}{p(C=2)}$ | (S1) |
| --- | --- | --- |

The observer knows that the measurement x is caused by the stimulus s, but has no knowledge of s. Therefore, the optimal observer marginalizes over s:

|  | $p\left( x \right\vert C)=\int p\left( x \right\vert s)p\left( s \right\vert C) ds.$ | (S2) |
| --- | --- | --- |

We substitute the expression for the measurement distribution and the generating category distribution, and evaluate the integral:

|  | $p\left( x \right\vert C)=\int N\left( s;x, \sigma^{2} \right) N\left( s;\mu_{C}, \sigma_{C}^{2} \right) ds=N(x; \mu_{C},{\sigma^{2}+ \sigma}_{C}^{2}).$ | (S3) |
| --- | --- | --- |

Using the task- and category-specific $\mu_{C}$and $\sigma_{C}$ in **Equation S3**, and substituting the resulting expression back into **Equation S1**, we get **Equation 6** and **Equation 7**.
