# Supplementary material for: Modality independent or modality specific? Common computations underlie confidence judgements in visual and auditory decisions
Source: PLoS Comput Biol. 2023 Jul 14;19(7):e1011245. doi: 10.1371/journal.pcbi.1011245 (PMC10426961; doi:10.1371/journal.pcbi.1011245)
Supplement: S9 Text — (DOCX) [file pcbi.1011245.s009.docx]

**S9 Text: Model Fitting**

Models were initialised with random starting values sampled from a normal distribution which spanned a reasonable and sufficiently large range, given the parameters of each model. To mitigate against the possibility of finding parameter estimates that corresponded to a local minimum in the parameter space, we used between 10-20 random starting values for each model and confirmed convergence of different starting values to similar best-fitting parameter estimates. The same set of starting values were used for each subject in each modality, where possible.
