# Supplementary material for: Modality independent or modality specific? Common computations underlie confidence judgements in visual and auditory decisions
Source: PLoS Comput Biol. 2023 Jul 14;19(7):e1011245. doi: 10.1371/journal.pcbi.1011245 (PMC10426961; doi:10.1371/journal.pcbi.1011245)
Supplement: S1 Table — (DOCX) [file pcbi.1011245.s010.docx]

S1 Table

*Categorisation Accuracy for Highest Intensity Stimuli and Exclusions*

|  | Task-Modality Configurations | | | |
| --- | --- | --- | --- | --- |
| Participant | Visual Different Means | Auditory Different Means | Visual Different SDs | Auditory Different SDs |
| 1 | 73.89 | 86.11 | 87.78 | 83.33 |
| 2^a^ | 78.33 | 62.22 | 80.56 | 56.67 |
| 3 | 90 | 83.33 | 82.78 | 81.67 |
| 4 | 94.44 | 82.78 | 86.67 | 85.56 |
| 5 | 89.44 | 80.56 | 78.89 | 76.11 |
| 6^b^ | 81.67 | 78.33 | 77.78 | 63.33 |
| 7 | 89.44 | 85.56 | 83.89 | 71.11 |
| 8 | 89.44 | 77.78 | 83.33 | 62.22 |
| 9 | 86.67 | 86.11 | 80.56 | 80.56 |
| 10^c^ | 71.67 | - | 73.33 | - |
| 11^d^ | 86.11 | 60.56 | 74.44 | **53.33** |
| 12 | 89.44 | 91.11 | 85.56 | 80.56 |

*Note.* ^a^ excluded for reported confusion about task instructions; ^b^ excluded for only using highest confidence rating in one session; ^c^ excluded for not completing all sessions; ^d^ excluded because categorisation accuracy was not significantly different from chance using a binomial test of significance (*p* > 0.05 where bolded in table).
