# Supplementary material for: Modality independent or modality specific? Common computations underlie confidence judgements in visual and auditory decisions
Source: PLoS Comput Biol. 2023 Jul 14;19(7):e1011245. doi: 10.1371/journal.pcbi.1011245 (PMC10426961; doi:10.1371/journal.pcbi.1011245)
Supplement: S2 Table — (DOCX) [file pcbi.1011245.s011.docx]

S2 Table

*Model-Free GLMM Follow-Up Tests: The Effect of the Evidence Predictor at all Intensity Levels*

| GLMM Evidence Predictor | |  | | Task-Modality Configurations | | | | | |  | | | | | |
| --- | --- | --- | --- | --- | --- | --- | --- | --- | --- | --- | --- | --- | --- | --- | --- |
|  |  | Visual Different Means | | Auditory Different Means | | | Visual Different SDs | | | Auditory Different SDS | | | | | |
|  | Category | | | | | | | | | | | | | |  |
| Intensity 1 | Odds Ratio | | 1.32*** | | 1.00 | | | 1.18** | | | | 1.00 | | |  |
|  | CI_lower_ | | 1.14 | | 0.85 | | | 1.05 | | | | 0.84 | | |  |
|  | CI_upper_ | | 1.52 | | 1.18 | | | 1.34 | | | | 1.18 | | |  |
| \| Intensity 2 \| \| --- \| \| | Odds Ratio | | 3.05*** | | 1.13 | | | 1.89*** | | | | 1.09 | | |  |
|  | CI_lower_ | | 2.31 | | 0.95 | | | 1.44 | | | | 0.89 | | |  |
|  | CI_upper_ | | 4.02 | | 1.34 | | | 2.48 | | | | 1.33 | | |  |
| \| Intensity 3 \| \| --- \| \| | Odds Ratio | | 7.33*** | | 10.62*** | | | 4.26*** | | | | 4.04*** | | |  |
|  | CI_lower_ | | 4.77 | | 5.91 | | | 3.29 | | | | 2.46 | | |  |
|  | CI_upper_ | | 11.25 | | 19.08 | | | 5.50 | | | | 6.62 | | |  |
| Intensity 4 | Odds Ratio | | 30.69*** | | 19.56*** | | | 15.36*** | | | | 5.69*** | | |  |
|  | CI_lower_ | | 12.67 | | 9.29 | | | 10.24 | | | | 3.03 | | |  |
|  | CI_upper_ | | 74.34 | | 41.19 | | | 23.05 | | | | 10.67 | | |  |
|  |  | | Confidence | | | | | | | | | | |  |  |
| Intensity 1 | Estimate | | 0.01 | | | 0.01 | | | 0.03 | | 0.00 | |  |  |  |
|  | CI_lower_ | | -0.03 | | | -0.01 | | | -0.01 | | -0.02 | |  |  |  |
|  | CI_upper_ | | 0.04 | | | 0.03 | | | 0.07 | | 0.01 | |  |  |  |
| \| Intensity 2 \| \| --- \| \| | Estimate | | 0.11*** | | | 0.02 | | | 0.05* | | 0.01 | |  |  |  |
|  | CI_lower_ | | 0.06 | | | -0.03 | | | 0.01 | | -0.01 | |  |  |  |
|  | CI_upper_ | | 0.16 | | | 0.07 | | | 0.09 | | 0.03 | |  |  |  |
| \| Intensity 3 \| \| --- \| \| | Estimate | | 0.23*** | | | 0.25*** | | | 0.19*** | | 0.25*** | |  |  |  |
|  | CI_lower_ | | 0.15 | | | 0.17 | | | 0.13 | | 0.14 | |  |  |  |
|  | CI_upper_ | | 0.31 | | | 0.34 | | | 0.25 | | 0.36 | |  |  |  |
| Intensity 4 | Estimate | | 0.43*** | | | 0.26*** | | | 0.30*** | | 0.25*** | |  |  |  |
|  | CI_lower_ | | 0.29 | | | 0.16 | | | 0.23 | | 0.13 | |  |  |  |
|  | CI_upper_ | | 0.56 | | | 0.36 | | | 0.37 | | 0.37 | |  |  |  |

*Note.* CI terms refer to 95% confidence intervals calculated using the profile likelihood method. Significance values are obtained using the Satterthwaite approximation to calculate the degrees of freedom for the t-distribution based on the estimated variance-covariance matrix of the model parameters [1]. For category GLMMs (top section), we report the odds ratio (exponentiated coefficient estimate) for ease of interpretation. The odds ratio represents the change in odds of the outcome for a one-unit change in the predictor variable. For confidence GLMMs (bottom section), we report standardised regression coefficients.

**p* < 0.050, ***p* < 0.010, ****p* < 0.001

**References**

1. Lüdecke D. _sjPlot: Data Visualization for Statistics in Social Science_. R package version 2.8.12. 2022. Available: <https://CRAN.R-project.org/package=sjPlot>
