# Supplementary material for: Modality independent or modality specific? Common computations underlie confidence judgements in visual and auditory decisions
Source: PLoS Comput Biol. 2023 Jul 14;19(7):e1011245. doi: 10.1371/journal.pcbi.1011245 (PMC10426961; doi:10.1371/journal.pcbi.1011245)
Supplement: Attachment — Submitted filename: Response_to_Reviewers_Common_Computations_Manuscript_v6.pdf [file pcbi.1011245.s015.pdf]

15 May 2023

THE UNIVERSITY OF QUEENSLAND
AUSTRALIA

Dear Associate Professor Christoph Mathys and Professor Thomas Serre,

Thank you for the opportunity to revise our manuscript entitled "Modality Specific or Modality Independent? Common Computations Underlie Confidence Judgements for Visual and Auditory Decisions". The reviewer comments were extremely insightful, and we have attempted to address them all in our revision. We believe the manuscript is much improved in light of the reviewer comments, and we hope it is now sufficient for publication in *PLOS Computational Biology*.

Thank you for your consideration,

Rebecca West
William Harrison, Natasha Matthews, Jason Mattingley and David Sewell

---

Reviewer comments are copied below in italics. Our responses are inset and in bold. Passages from the revised manuscript are further inset, italicised, and corresponding line numbers are given.

Reviewer #1 Comments:

The authors investigate through modeling fitting several computational models for confidence judgements, and whether they generalize to both visual and auditory tasks. The authors find that scaled evidence strength models outperform unscaled and Bayesian models. The paper builds over previous literature on similar experiments but adds novelty by introducing the study of two modalities and an exhaustive model comparison which is of value.

1) My main criticism on this paper is at the interpretative side: the authors seem to imply, based on the model fitting, that humans are non-Bayesian. But this conclusion totally depends on the modelling assumptions. I think that the authors make a good job in comparing different models quite exhaustively but concluding that "it is unlikely that confidence judgements…. are generated by a Bayesian computational process" can be too much of a stretch. This is so because things like priors in the Bayesian model, or different noise models (e.g., non Gaussian) could account for the departures of the model with the data (see the relevant paper of Schustek et al, Nature Comm, 2019).

> **We thank Reviewer #1 for their feedback on the interpretative side of our manuscript. We have made several changes in the revised manuscript to address this criticism and soften our conclusions about Bayesian models.**
>
> **"Although observers take sensory uncertainty into account, they do not appear to do so in a way that is consistent with Bayesian models of confidence in either modality" has been revised as:**
>
> > *Although we found consistent evidence that observers take sensory uncertainty into account, we observed significant differences between our empirical data and the predictions of the Bayesian models.* [Lines 1202-1204]

We have also added a limitations section to the Discussion in the revised manuscript which notes some of Reviewer #1's concerns about the Bayesian models:

> *Although our study used a highly representative set of Bayesian models from the broader confidence literature, we could not feasibly test the full space of possible models that might be considered "Bayesian". We, therefore, cannot rule out the entire Bayesian class of confidence models completely. In particular, our Bayesian models may have been limited by only incorporating a point estimate of sensory uncertainty, rather than a distribution over uncertainty (Boundy-Singer et al., 2022). Furthermore, we did not consider models with non-Gaussian noise, lapse rate parameters or non-Gaussian priors. Our findings, nonetheless, strongly suggest confidence judgements in the visual and auditory modalities are unlikely to derive from posterior probability computations as defined by the set of Bayesian models considered here. Thus, although Bayesian models are particularly powerful for their generalisability, that is they involve the computation of a probability metric which can be compared directly across domains, their poor fit to empirical data highlights the need to explore non-Bayesian frameworks that can better capture human behaviour in different contexts. Our study, in particular, demonstrates that non-Bayesian algorithms such as the scaled evidence strength models can operate on standardised units and provide a powerful account of human confidence judgements across modalities.* [Lines 1240-1256]

2) Also, the scaled model is not mechanistic nor normative, so while Bayesian models could be taken to generalize to other domains, the mechanistic model might ***[not]*** *be able to do so. I think that the authors should restate the implications of their results in view of this criticism* and *answer the question of what could be the reason for having scaling evidence strength with linear of quadratic dependences.*

We understand Reviewer #1's comment as relating to the generalisability of the Bayesian and scaled evidence strength models across domains. The direct generalisability of the Bayesian models is clear as they assume the computation of a probability metric, which is a unit of measurement that can be directly compared across domains. We disagree, however, that the same principles do not apply to the scaled evidence strength models. We believe that the scaled evidence strength models are mechanistic at a cognitive level of analysis and, as demonstrated by our study, capable of generalising across domains. Our study suggests that by assuming that the scaling mechanism operates on standardised units, we can implement the algorithm across a range of different decisional contexts.

In our study, the standardisation of sensory units is consistent with the idea that observers built up a good internal representation of the categories during training. The z-score captures this sense of understanding the relative frequencies of different stimulus values within each category distribution. Beyond sensory modalities though, the scaled evidence strength models could operate on a range of different standardised units. To illustrate, for value-based decision-making, utility can be normalised for a given task context in the same way as a distribution of sensory information can be. In the revised manuscript, we have added some additional commentary to highlight our thoughts on this issue:

Although our tasks were carefully designed to allow for comparison across modalities, we believe that the scaled evidence strength class of models can generalise beyond the narrow conditions investigated here. In our tasks, the decision variable for the scaled evidence strength models was operationalised as a noisy measurement of the stimulus feature of interest (standardised orientation or frequency). For other tasks, however, the decision variable may represent a more complex, multi-dimensional signal. For example, the decision variable could represent sensory information that has been integrated across domains (i.e., an audio-visual signal), utility in a value-based decision-making task or any other normalised variable in a range of decision-making tasks. Our results strongly suggest that this class of models provides a promising direction for future research to investigate a generalisable algorithm for confidence. [Lines 1310-1321]

As we indicate in the manuscript, investigating how observers might use such algorithms when integrating sensory signals across modalities (e.g., an audio-visual task) or comparing confidence across domains is an important avenue for further research.

Based on Reviewer #1's comment, in the revised manuscript, we also wanted to provide some additional commentary on the linear and quadratic scaling of the boundaries in the scaled evidence strength class. We have added the following to the Discussion:

Although we found that observers use sensory uncertainty-dependent boundaries for category and confidence judgements, the specific way sensory uncertainty scales the boundaries was not universal across modalities or tasks. Within the scaled evidence strength class, we tested different assumptions about the scaling rule that was used to adjust the confidence criteria. We compared models which used either a linear ($k + m\sigma$), quadratic ($k + m\sigma^2$), or free-exponent ($k + m\sigma^a$) scaling rule. We estimated a value of k and m for each boundary, which allowed for the strength of the effect of sensory uncertainty on the positioning of the boundaries to differ across response options (i.e., in the different means tasks, the confidence 4 boundary could shift more than the confidence 1 boundary). In the case of the linear scaling rule, m, was a constant that was applied to the estimate of sensory uncertainty which determined the magnitude of the sensory-uncertainty-based shift in choice/confidence boundaries. In the cases of the non-linear scaling rules, an exponent was also used on the estimate of sensory uncertainty assuming that the impact of sensory uncertainty on confidence criteria was not proportional to the amount of sensory uncertainty. Rather, these models assumed that the impact of sensory uncertainty became more pronounced at higher levels. These different models allowed us to compare the relative importance of different scaling rules which quantified different assumptions about the effect of sensory uncertainty on choice/confidence criteria. We found, however, that both the linear and non-linear scaling rules accounted for the empirical data well and we did not find consistent evidence in favour of either alternative.

The lack of consistency within the scaled evidence strength class suggests that the scaling of sensory uncertainty itself is important, but that the relative

importance of different scaling factors differs across different contextual factors such as modalities, tasks and individuals. [Lines 1275-1299]

We believe that the above changes to the manuscript, which address the generalisability of the scaled evidence strength models and purpose of the linear/quadratic scaling of the boundaries, provide substantial clarity about the underlying computational mechanisms of the model classes and we are grateful for Reviewer #1's feedback on these issues.

3) Second, the models assume that there is a point estimate of uncertainty. But it is probably that in the Bayesian model agents need to consider also a distribution over uncertainty. Such a distribution over uncertainty could be an additional free parameter that could effectively change the Gaussian noise distribution to another one with a longer tail. Does such a model make a better job in fitting the data?

We liked this suggestion from Reviewer #1. Following their recommendation, we tried a version of the Bayesian model that assumed a distribution over uncertainty, rather than a point estimate. We found, however, that the critical uncertainty parameter in this model recovered poorly and we therefore did not include it in the revised manuscript. We provide a description of the model we explored in response to this comment from Reviewer #1 and the associated amendments to the manuscript below.

Following Boundy-Singer, Ziemba and Goris (2022), we assumed that the distribution of uncertainty follows a log-normal distribution because any single estimate of uncertainty cannot fall below zero. The mean of the uncertainty distribution was estimated as a free parameter for each stimulus intensity level, (i.e., the parameters $\sigma_1 - \sigma_4$ in the original manuscript). The standard deviation of the uncertainty distribution was also estimated as a free parameter, σ_U, but was assumed to be constant across stimulus intensity levels (Boundy-Singer et al., 2022). To estimate the standard deviation of the uncertainty distribution, for each trial we took 100 samples from the log normal distribution, with the defined mean and standard deviation, in steps of constant cumulative density (from 1%, 2%, …, 99%, 100%). We used each sample to compute a response probability using Equation 7 (in the revised manuscript). We then calculated the weighted sum of these probabilities using the normalised densities for each draw of σ. In effect, this model allowed for an observer's internal representation of the stimulus to be associated with a distribution of sensory uncertainty. We performed a parameter recovery analysis for this model and found that the uncertainty parameter (labelled as *sig_sd*) did not recover well:

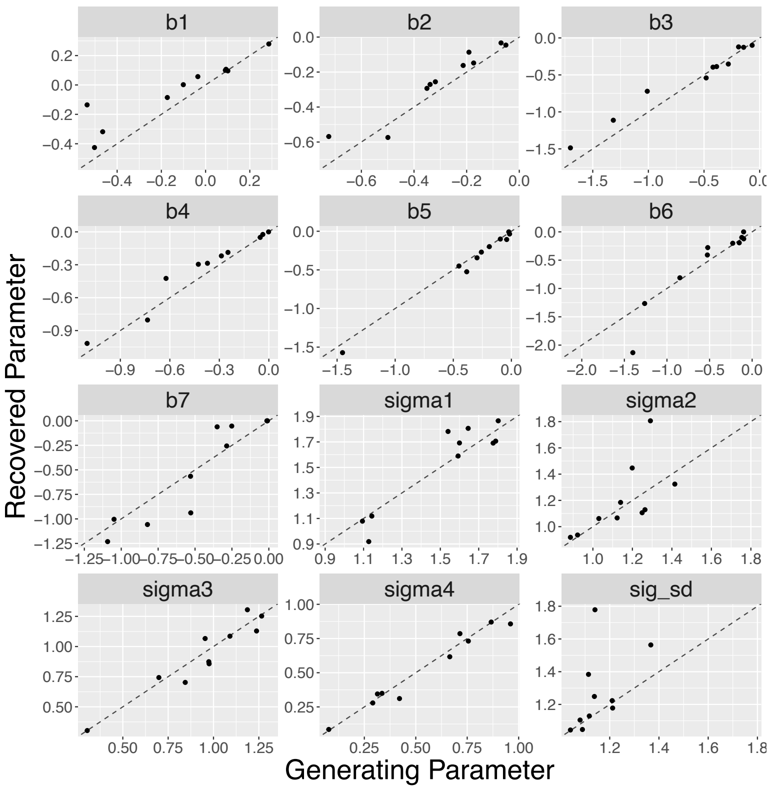

Given the relatively poor recovery of the parameter of interest, we suspect that this variation of the Bayesian model warrants further consideration, but likely requires a different task or model architecture. In light of this, rather than include the model itself in the revised manuscript, in the Discussion we have included this as a limitation and an avenue for further investigation:

> *In particular, our Bayesian models may have been limited by only incorporating a point estimate of sensory uncertainty, rather than a distribution over uncertainty (Boundy-Singer et al., 2022)* [Lines 1243 - 1245]

4) In the list of refs in line 155, the paper by Moreno, Neural Computation, 2010 on confidence judgments is missing. Also, the paper by Drugowitsch lab, Drugowitsch et al, J of Neuroscience, 2012, seems to be relevant and earlier to those quoted.

We thank Reviewer #1 for pointing out these references and we have now included both of them in the revised manuscript on lines 182-183 and lines 1324-1325.

5) Regarding the first paper, do the authors recorded the reaction times? If yes, one could model drift-diffusion models to the data. The Bayesian models does not only predict how accuracy and confidence depends on the stimulus strength (drift rate) and uncertainty (inverse noise variance), but also on the reaction time. A discussion of these effects could be relevant if the data are available, or in the Discussion if they are not available in the current experiment.

We thank Reviewer #1 for commenting on response times because we agree that this is an important topic which we neglected to mention in the original manuscript. We did record response times. We are, however, hesitant to model our response time data with evidence accumulation/ diffusion models for several reasons.

Our experimental paradigm was not designed to precisely capture variations in response time. Our design notably emphasised accuracy over speed. We only provided feedback on the correctness of responses, both during training and at the end of experimental blocks during testing. In fact, participants were not given any explicit instructions about the speed of their responses. We, therefore, do not think that our response time data would be appropriate for a meaningful investigation of evidence accumulation models. However, we acknowledge that designing a study to specifically incorporate response times into the analysis is an important goal for future research.

Furthermore, given the number of model variants in the original manuscript, we do not see an investigation of evidence accumulation models as feasible. This is because the class of evidence accumulation models includes many variants which make a range of different assumptions. Some of these assumptions include whether decision time is used to calculate the probability of being correct (Kiani et al., 2014; Kiani & Shadlen, 2009; van den Berg et al., 2016); whether the accumulators for different choice options are separate (Moreno-Bote, 2010; Vickers, 1979) or interact (Zylberberg et al., 2012); whether there is additional accumulation of evidence post choice (Balsdon et al., 2020; Pleskac & Busemeyer, 2010) or whether the confidence decision variable is accumulated directly (Ratcliff & Starns, 2009, 2013). In addition, if we were to investigate some of the above models and constrained them by our empirical RT distributions, it is not clear how we would compare the performance of these models to the SDT-based models in the original manuscript, given that they would be fit to different data.

Given the above considerations, we see the purpose of our study as narrowing the scope of possible models and assumptions that can be made about the perceptual and confidence decision process. A formal investigation of how this might be implemented in evidence accumulation models to account for choice, confidence and response time and how these models would apply across sensory modalities should be investigated in future research.

To address the above concerns directly, we have added the following paragraph to the Discussion in the revised manuscript:

> *Of note, we did not consider models which jointly account for choice, confidence, and response time. Given the established link between response time and confidence (Drugowitsch et al., 2012; Kiani et al., 2014; Maniscalco & Lau, 2016; Moreno-Bote, 2010; Pleskac & Busemeyer, 2010; Ratcliff & Starns, 2009, 2013; van den Berg et al., 2016), we see a formal investigation of the generalisability of response time-based models across domains as an important field of investigation for future research. Our study serves an important role in narrowing the scope of potential models and provides several important considerations for future studies investigating response time. In particular, our results show that in addition to the strength of the signal, the amount of sensory uncertainty in the signal plays an important role in the formation of confidence, beyond just adding noise to the decision-making process. This is true across different decisional domains, that is, different task structures and modalities.* [Lines 1322-1334]

6) Methodologically, it is convenient to use 4 confidence ratings for the model fitting, but it does not seem to be the most natural way of conveying confidence, which can be made

numerically by the participants using the notion of probability of being correct. It could be relevant to describe in the Methods the pros and cons of this choice.

In the revised manuscript, we have added the following to the Methods:

We chose to use a 4-point confidence rating scale as it is common in the study of confidence and allowed for meaningful comparison of models to previous research (Adler & Ma, 2018; Denison et al., 2018; see Tekin & Roediger, 2017 for investigation of alternative methods for collecting confidence ratings) [Lines 357-361]

7) While there is overlap of the category distributions in the visual task, in the auditory task the overlap seems to be minor (comparing the mean distance and the standard deviations). It is unclear the reason for this choice, so a clarification could be beneficial.

We believe that Reviewer #1 may be conflating the auditory and visual modalities with the Different Means and Different SDs tasks. These factors were factorially combined, so both the auditory and visual tasks had versions where category overlap was relatively low (Different Means – top panel in figure below) and relatively high (Different SDs – bottom panel in figure below).

The overlap of the category distributions in the visual tasks and the auditory tasks is approximately the same, as shown in the Figure below. Compare left panels (visual) with right panels (auditory).

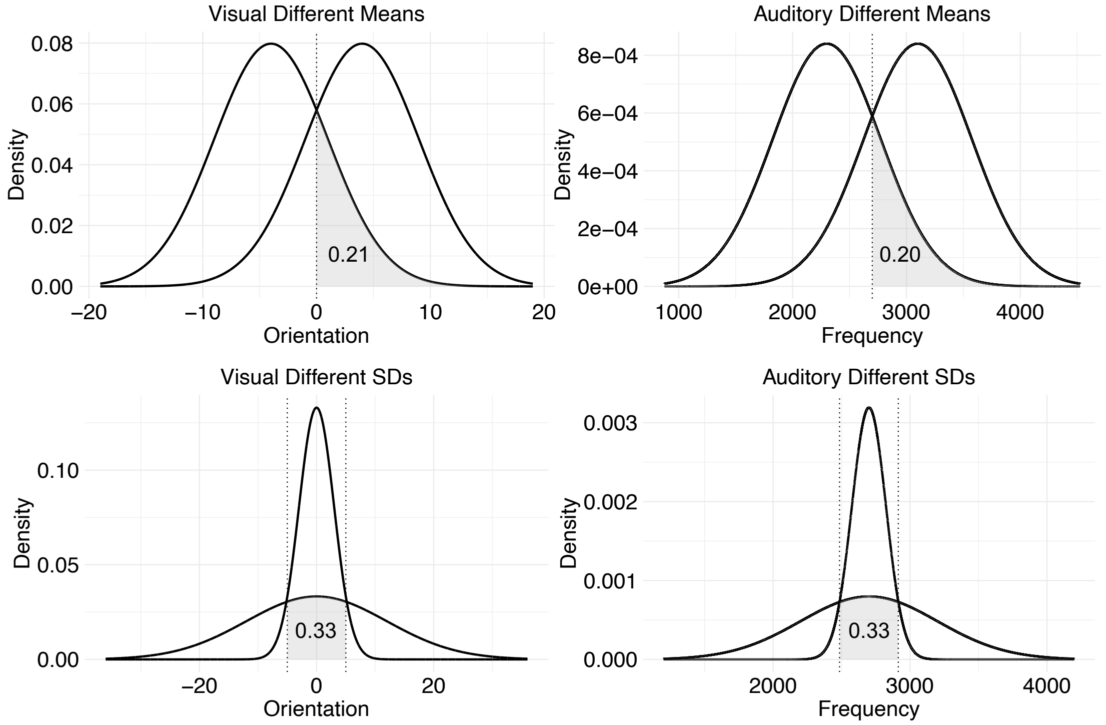

In the above figure, we confirmed this analytically for all task-modality configurations by defining two normal distributions according to the parameters of the category distribution for a given configuration, *cat1* and *cat2*. We then found the points at which the two distributions intersect by finding all roots of the function *cat1*-*cat2*. For the different means tasks, the distributions intersect at a single point, depicted as a vertical dotted line in the figure above. For the different SDs tasks, the distributions intersect at two different points, depicted as two vertical dotted lines in the figure above. We then calculated the area of

overlap between the two distributions by integrating the function pmin(*cat1*(*x*), *cat2*(*x*)). For the different means tasks, we calculate the area of overlap between the single intersection point and infinity. For the different SDs tasks, we calculate the area of overlap between the first intersection point and the second intersection point. As shown in the Figure above, this approach demonstrates that the amount of overlap between the category distributions in the visual and auditory tasks is approximately equal. The figure also demonstrates that the overlap of the distributions is greater for the different SDs tasks than the different means tasks.

8) In line 391, it is unclear whether b1 lies in between b2 and -b2, or can be independently fitted.

We understand Reviewer #1's comment as indicating that it is not clear whether *b2* and *-b2* were positioned symmetrically around some midpoint (and *b1* was fit independently) or whether *-b2* and *b2* were symmetrical around *b1*. We appreciate that this was a legitimate source of confusion in the original manuscript.

For the different means task, we constrained *b1* to be between *-b2* and *b2*. To make this more clear, in the revised manuscript we have removed a sentence which stated 'that two measurements that are equidistant from the intersection of the category response region (and therefore should have the same evidence strength) should logically have the same confidence associated with them'. We appreciate that if *b1* is biased in either direction from the true overlap in the category distributions, stimulus values that are equidistant from *b1* will NOT have the same evidence strength. We have also included some more detail in the revised manuscript to further clarify the constraint on the positions of the boundaries:

> *The boundaries in the different means task were ordered such that:* $-b_4 < -b_3 < -b_2 < b_1 < b_2 < b_3 < b_4$*, where the position of each boundary was freely estimated, but constrained by monotonicity.* [Lines 452 – 454]

9) In Fig. 4, it is unclear what point corresponds to. Is it an average across participants? It would be important to clarify this, and maybe also add error bars.

In the revised manuscript, we have updated the Figure 5 caption to clarify what the points correspond to. We have also added error bars to the Figure:

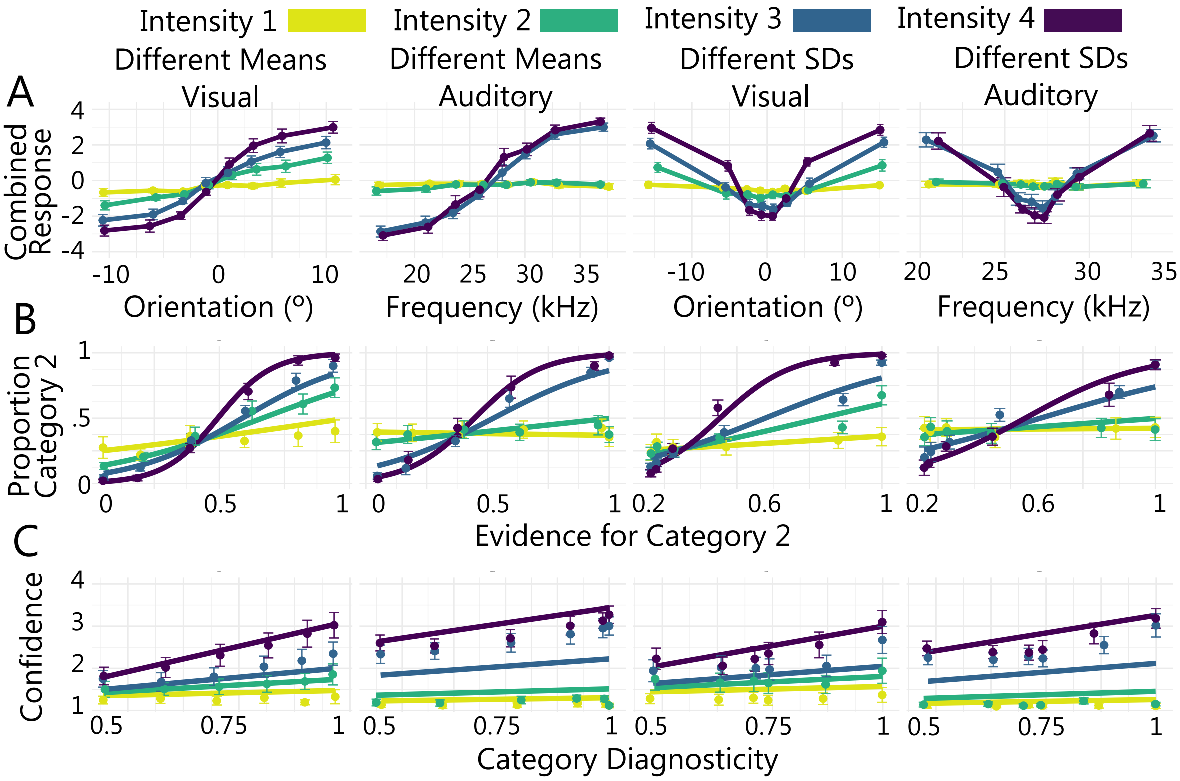

Fig 5. Logistic and Linear Mixed Models. *(A) Mean combined category and confidence response across all participants, where value indicates confidence and polarity indicates category, as a function of binned stimulus value. (B) Model-free GLMM. Category decisions as a function of category 2 evidence. Data points show empirical data. Solid lines show logistic model predictions for the fixed effect of evidence at each intensity level. (C) Model-free GLMM. Confidence as a function of category diagnosticity. Data points show empirical data. Solid lines show linear model predictions for the fixed effect of category diagnosticity at each intensity level. Error bars show ±1 SEM. Where error bars are not visible, they are occluded by the plotting symbols.*

10) In the author summary, I would more clearly define "a single class of models", and "governed by the same general process". This wording is largely ambiguous and unspecific.

We thank Reviewer #1 for the feedback about ambiguity in some of the wording in the author summary. We have revised the author summary to be more specific about the role of the model classes and our conclusions about the 'same general process'. The new summary reads:

> *In this study, we investigated the computational processes that describe how people derive a sense of confidence in their decisions. In particular, we used computational models to describe how decision confidence is generated from different stimulus features, specifically evidence strength and sensory uncertainty, and determined whether the same computations generalise to both visual and auditory decisions. We tested a range of different computational models from three distinct theoretical classes, where each class of models instantiated different algorithmic hypotheses about the computations that are used to generate confidence. We found that a single class of models, in which confidence is derived from a subjective assessment*

of the strength of the evidence for a particular choice scaled by an estimate of sensory uncertainty, provided the best account of confidence for both visual and auditory decisions. Our findings suggest that the same type of algorithm is used for evaluating confidence across sensory modalities but that the 'settings' (or parameters) of this process are fine-tuned within each modality. [Lines 33-47]

We hope that this addresses Reviewer #1's concerns about ambiguity while remaining a non-technical summary of the work, as per the *PLOS Computational Biology* Submission Guidelines.

11) The terminology "response" in Fig. 4 can be confusing, as it adds to confidence and category responses, used as well.

In the revised manuscript, we have updated the terminology to 'combined category and confidence response'. [Line 715]

Minor comments:

12) Some papers listed in the paper do not show in the reference list, as far as I can tell.

We thank Reviewer #1 for drawing our attention to this and we apologise that we overlooked some papers in the reference list. We have now crossed check the intext references and reference list in the revised manuscript.

13) There seems to be some problem in the pdf version with how the gratings are displayed in Fig. 1A and 1B.

We are not sure what the specific issue that Reviewer #1 encountered was. We do intentionally depict a low contrast grating (on the right) in Figure 1B to illustrate conditions under which there may be greater noise in the observer's internal representation of the stimulus.

In case the depiction of the low contrast grating was the source of confusion, in the revised manuscript we have added some additional text to the Figure caption:

In this example, the probability distributions depict noisy representations of the orientation of a Gabor patch. Evidence strength varies with distance from the decision criterion and sensory uncertainty with the contrast of the Gabor. [Lines 114-116]

14) In line 501, redefining d could add clarity in this passage.

We interpret Reviewer #1's comment as indicating we should provide additional clarity about the Bayesian decision rule. In the revised manuscript, we have added the following text to clarify what *d* represents:

When the perceived value of a stimulus on a given trial is x, we assume that the observer uses the log posterior probability ratio to make a decision. The log posterior probability ratio represents the (log) ratio of posterior beliefs about category membership, given the properties of the stimulus, such that $d = log\frac{p(C=1 \mid x)}{p(C=2 \mid x)}$*. When the log posterior probability ratio is positive, there is greater evidence for category 1.* [Lines 567-571]

We have also included the details of the derivation of *d* in the revised Supplementary Information.

Derivation of d for Different Means and Different SDs Tasks

Following Adler and Ma (2018), we used Equation 4 and Equation 5 for the computation of d. We provide the details for derivation of d below, in the same way as they are provided in Adler and Ma's original paper.

The log posterior ratio is equivalent to the log likelihood ratio plus the log prior ratio:

$$d = log\frac{p(C = 1 \mid x)}{p(C = 2 \mid x)} = log\frac{p(x \mid C = 1)}{p(x \mid C = 2)} + log\frac{p(C = 1)}{p(C = 2)} \quad \text{(S1)}$$

The observer knows that the measurement x is caused by the stimulus s, but has no knowledge of s. Therefore, the optimal observer marginalizes over s:

$$p(x \mid C) = \int p(x \mid s)\, p(s \mid C)\, ds. \quad \text{(S2)}$$

We substitute the expression for the measurement distribution and the generating category distribution, and evaluate the integral:

$$p(x \mid C) = \int N(s; x, \sigma^2)\, N(s; \mu_C, \sigma_C^2)\, ds = N(x;\ \mu_C, \sigma^2 +\ \sigma_C^2). \quad \text{(S3)}$$

Using the task- and category-specific μ_C *and* σ_C *into Equation S3, and substituting the resulting expression back into Equation S1, we get Equation 4 and Equation 5.* [Lines 172-183]

Reviewer #2 Comments:

This is comprehensive and careful work examining the extent to which different computational models of confidence generalise across visual and auditory modalities. Such a computational approach has great promise to shed light on the extent to which metacognition is domain-general or domain-specific, which has been much debated in the literature but has often only focused on summary statistics such as meta-d' rather than a detailed consideration of the model that generates confidence data. Here, this is tackled with 2 complementary tasks that were matched across modalities, and in-depth psychophysical characterisation of confidence thresholds across 8 observers.

I am enthusiastic about this paper which I think should be published following revisions. I list a number of specific comments below.

1) The analysis and model comparison is comprehensive, and I appreciate the careful work that has gone into the parameter recovery simulations reported in the Supplement. However, at a number of points in the manuscript I found myself wondering how identifiable a specific model is. For instance, the scaled evidence-quadratic and Bayesian models in Figure 3 look very similar in terms of their predictions for confidence thresholds. Model recovery can be easily established using a ground-truth simulation, in which data from each model is simulated and then fit with each candidate model, to generate a confusion matrix (see eg https://elifesciences.org/articles/49547).

> **We thank Reviewer #2 for this suggestion and agree about the importance of a model recovery analysis. In the revised manuscript, we have included the model recovery results in a similar format to that shown in the Wilson and Collins (2012) paper provided by Reviewer #2. In the main text, we describe our model recovery procedure and include a figure showing the recovery result for a representative model from each class:**
>
> > *We also performed a model recovery analysis to test our ability to distinguish our core models and confirm our model-comparison results. For each model, we generated 100 synthetic datasets by simulating responses to a set of randomly sampled stimulus values that were similar to the empirical stimulus data. We simulated responses to these stimuli using randomly sampled parameter values that were similar to those obtained through fitting participant data. Each dataset contained 720 simulated 'trials', matching the size of the empirical dataset from each participant in the main experiment. We then fit all models to every dataset and calculated the probability that the model used to generate the data was the best fitting model, according to a given model selection metric, across all datasets. In Fig 9A, we show the model recovery results for a representative model from each class. We show the full details of model recovery using both AIC and BIC for model selection for all models in the Supplementary Figure 2. As shown in Fig 9A, we found that across model classes, the simulated data were almost always best fit by the model that generated them. This result indicated that the model classes were distinguishable from one another and confirmed our model comparison results.* [Lines 1131 - 1146]

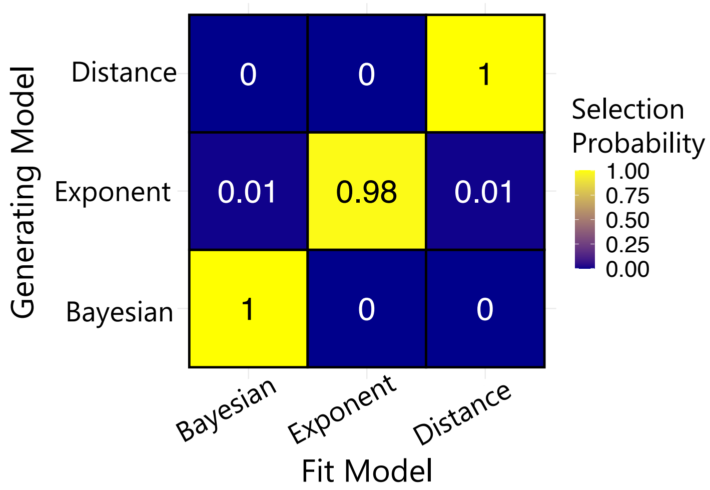

Fig 9. Model Recovery. *Numbers and colours denote the probability that the data generated with model X (x-axis) are best fit by model Y (y-axis). Selection probabilities were calculated using AIC for model selection. (A) Confusability matrix for core models using a representative model from each model class.*

In the Supplementary Material, we have included the model recovery results for all models (not just a representative model from each class). We provide the confusability matrix for selection probabilities when using both *AIC* and *BIC* for model selection. We also provide some commentary on model confusability within model classes which can be found on Lines 190 – 206 in the Supplementary Material. We found that, as expected, there was some confusability within model classes. The free-exponent model, for example, is a generalisation of the linear and quadratic models that includes the latter two as special cases. Thus, one would expect the recovery for the free-exponent model to be poorer the closer the generating value of the exponent approaches either 1 (linear) or 2 (quadratic). Importantly, however, we did not find much confusability across the model classes where the unscaled evidence strength, scaled evidence strength and Bayesian models were distinguishable from one another using both *AIC* and *BIC* for model selection.

In addition, it seems important to establish to what extent a model in which parameters are shared / flexible across two candidate modalities can be accurately recovered, as a worry in the last section is that the domain-specific model is preferred due to this additional flexibility.

In the revised manuscript, we have included the following to address this comment:

We also performed a model recovery analysis for the models used to evaluate the parameter settings across modalities. In Fig 9B, we show the results from these models, where we again found that the simulated data were almost always best fit by the model that generated them. See Supplementary Figure 3 for full details of model recovery. This model recovery validated the model comparison

results that were used to compare the parameter settings of the free-exponent model across the visual and auditory modalities (see Fig 8). [Lines 1149-1155]

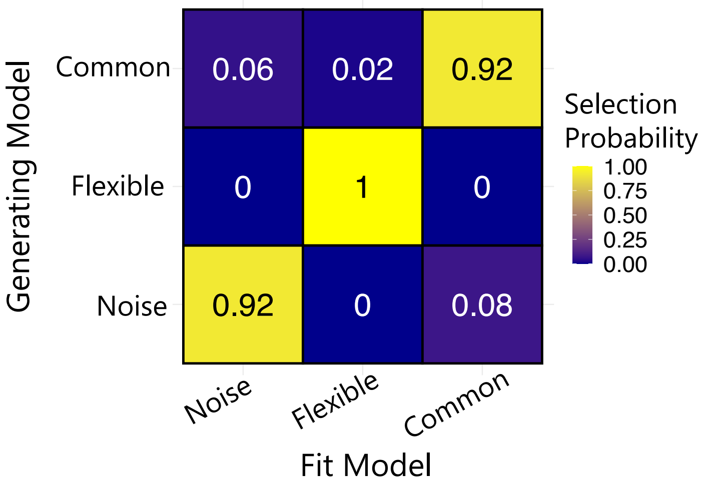

Fig 9. Model Recovery. *Numbers and colours denote the probability that the data generated with model X (x-axis) are best fit by model Y (y-axis). Selection probabilities were calculated using AIC for model selection. … (B) Confusability matrix for models used to compare parameter settings across modalities.*

We also provide parameter recoveries for these models in the revised Supplementary Material as they were not included in our original submission. These can be found in Supplementary Figures 14-16.

We would like to note that in the revised manuscript we removed two of the models from this section: the common boundaries (offset noise) and common boundaries (scaled noise) models. We chose to remove these models to simplify both the parameter recovery and model comparison results. Both of these models could be captured by the different noise model (albeit with a greater number of parameters) and therefore, we did not believe that they contributed substantially to our theoretical understanding of how the cognitive computations used for confidence generalise across vision and audition.

2) In Table 1, the model-free GLM, were there significant interactions of predictors with task? Such an analysis would provide a model-free way to ask if performance and/or confidence profiles differed between the two modalities, prior to fitting of candidate models.

We appreciate this astute suggestion from Reviewer #2 and have provided the details of this analysis in the Supplementary Materials [Lines 41 – 163].

3) Is there a reason that the different-means task for audition exhibits apparently binary dependence on intensity (eg in Figure 5)? It looks like intensity values 1/2 and 3/4 cluster together, whereas in vision the influence of intensity is more graded.

We thank Reviewer #2 for their comment as we agree that we did not provide sufficient commentary on this result in the original manuscript.

As noted, we observed a clustering of the effect of intensity in the auditory modality for both the different means and the different SDs task (although it's less obvious in Figure 5 for the different SDs task). Despite substantial pilot testing, we believe these results are due to the fact that we did not choose

sufficiently distinguishable differences in the intensity levels for the auditory stimuli. We, therefore, ran a small control study in 4 participants (who participated in the main experiment), where we used different intensity levels. In the control study, we observed the same graded effects of stimulus intensity in the auditory modality, as in the visual modality in the main experiment. We chose to repeat the experiment with the different SDs task, as opposed to the different means task, as this is where we observed the greatest differences in model performance. We confirmed the main model-based results with the control data. We did not report the results of this study in the main manuscript because of the small sample size. However, we do believe that this was an important exercise to investigate the nature of the differences across modalities.

In the revised manuscript, we have added the following text:

> *Of note, in the auditory modality the empirical data cluster across intensity levels, suggesting that participants did not appear to be able to distinguish between the two lowest intensity levels and the two highest intensity levels in the auditory modality in the same way as they did in the visual modality. The model predictions, however, do not capture this effect directly. We provide additional discussion of this feature of the data in the Supplementary Material (see Control Study including Supplementary Figure 18, Supplementary Table 9, Supplementary Figure 17, Supplementary Table 10, Supplementary Table 13).* [Lines 766 - 774]

4) On the potential limitations of Bayesian models – I wondered whether allowing for uncertainty over stimulus uncertainty would provide additional help here, especially given that part of the failure in the Bayesian model fits appears to be a miscalibration of confidence with respect to (performance-dependent) fitted uncertainty. I am not suggesting adding another model to the mix, but the authors might wish to consider this in Discussion, in relation to this paper: https://www.nature.com/articles/s41562-022-01464-x

We appreciate this suggestion from Reviewer #2 and are grateful for the reference that this reviewer provided. We received a similar suggestion from Reviewer #1 (see comment #3 in response to Reviewer #1, above) and as described above, we made some attempt to parameterise a Bayesian model which assumed a distribution over stimulus uncertainty, rather than a point estimate. The critical parameter in this model, however, did not appear to recover well in our model architecture. We, therefore, took Reviewer #2's suggestion and noted this limitation in the Discussion:

> *In particular, our Bayesian models may have been limited by only incorporating a point estimate of sensory uncertainty, rather than a distribution over uncertainty (Boundy-Singer et al., 2022)* [Lines 1243 - 1245]

Reviewer #3 Comments:

The manuscript is a report of four psychophysical experiments and extensive computational modelling of the ability of human participants to judge their confidence in the correctness of their perceptual decisions. The psychophysical experiments cover two binary categorical tasks, one where stimuli were drawn from two distributions that had different means but same variance, and the other where stimuli were drawn from two distributions that had same mean but different variances. These two tasks were run in both the visual and the auditory sensory modalities. The computational modelling covers three different classes of models, the first where the strength of their sensory evidence is taken directly to inform confidence evidence, the second where the sensory evidence is scaled by some factor that can depend on the intensity of the sensory signal, and the third based on a Bayesian account of the tasks. The authors find that the scaled evidence models were better models overall, for both sensory modalities, and especially for the different variances tasks. The manuscript is extremely well written, with an acute sense for clarity in the description of the results and models. The analyses are trustworthy and the comparison across tasks, sensory modalities, and model classes is particularly welcome in the growing literature on perceptual confidence. I have just a few comments.

First, I fail to understand how the unscaled evidence strength model can work across sensory modalities (see description on lines 438ff). Since the sensory evidence is a noisy bias-free estimate of the sensory stimulus (Equation 1, page 18), this evidence has the unit of the independent variable (i.e. degrees for the visual task, Hertz for the auditory task). So, how can one place the same perceptual boundaries 'b_r' for both the visual and the auditory tasks? The same question arises for the scaled evidence model with common settings (see lines 467ff). Was this possible because the participants data were normalized in some way? This may be what the authors call "standardised stimulus values" (page 38, line 763). I think the authors should try to clarify this point.

We agree with Reviewer #3 that this was not sufficiently clear in the original manuscript. We did standardise stimulus values and we have now added a paragraph of text to explain the standardisation process:

To directly compare stimulus values and estimated model parameters across modalities, we standardised stimulus values before model fitting. To standardise stimulus values, we subtracted the mean and divided by the standard deviation of all stimulus values in each domain such that:

$$Z_i = \frac{s_i - \mu_{s[M,T]}}{sd_{s[M,T]}} \qquad (4)$$

where M represents a given modality (visual or auditory) and T a given task (different means or different SDs); Z is the standardised stimulus value; s is the true stimulus value in perceptual units (degrees or hertz); μ_s is the mean of all presented stimulus values for that task and modality; and sd_s is the standard deviation of the same set of stimulus values. The standardisation of sensory units is consistent with the idea that participants built up a reliable internal representation of the categories during training such that they understood the relative frequencies of different stimulus values within each category distribution. [Lines 381 - 392]

We have also added a figure to show how this relates stimulus values across modalities. As a result of the standardisation, a given level of evidence for a

particular category corresponded to the same standardised stimulus value in both the visual and auditory modality. This meant that boundaries, which were estimated in standardised stimulus units, could be compared directly across modalities.

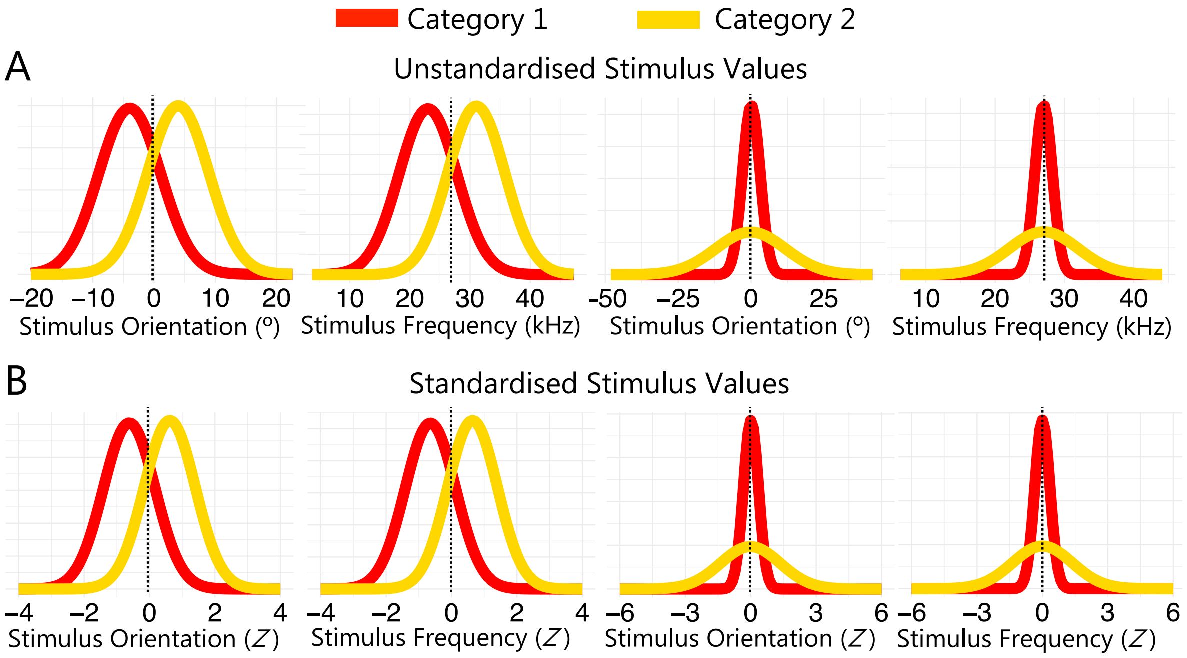

Fig 3. Presented Stimulus Values Before and After Standardisation for All Tasks. *(A) Presented stimulus orientations (visual modality) and stimulus frequencies (auditory modality) across different means (left panels) and different SDs (right panels) tasks before standardisation. Note similarity in presented stimulus distributions with true generating distributions in Fig 2. (B) Presented stimulus values after standardisation. Stimulus values were standardised by subtracting the mean and dividing by the standard deviation for all presented stimulus values, separately for each of the four task-modality configurations.*

Second, it is not clear to me how the evidence for one category and the category diagnosticity variables were computed (see Figures 4B and 4C). These variables are defined in Equations 12 and 13, but these equations are based on the knowledge of 'x', but my understanding is that 'x' is a noisy measurement at each trial (see page 18, line 342), and thus it is an unknown internal variable. Did the authors use the average noisy measurement consistent with the participant's categorical response and confidence judgment?

We thank Reviewer #3 for noting this inconsistency. Reviewer #3's understanding of *x* is correct and our inclusion of *x* in Equation 13 and 14 (equation numbers relate to those in the revised manuscript) was inaccurate. We have updated *x* (used to denote the observer's internal representation of the stimulus) to *s* (the actual value of the stimulus) in the relevant equations. We use the true value of the stimulus to calculate the 'evidence for category 2' and 'category diagnosticity' variables.

We agree with Reviewer #3 that using the average noisy measurement of the stimulus across participants would be interesting. We, however, chose to use the true value of the stimulus rather than a noisy measurement because the goal of this analysis was to determine if participants' responses aligned with the *true* amount of *evidence* associated with a particular stimulus value. In a sense, this

was a noise-free *'ideal observer model'*, designed to determine if participants understood the requirements of the different tasks and confirm that our chosen stimulus manipulations had a similar effect on responses across modalities. Our goal was to establish a sensible behavioural paradigm for which we use the computational models described in the 'Model Specification' section to estimate psychological constructs, such as noisy internal representations.

We hope that this provides appropriate context for our choice of variables in Equation 13 and 14 and we, again, thank Reviewer #3 for drawing our attention to the incorrect use of *x* in these Equations.

Third, I think it would be interesting to report the values of some of the fitted parameters, at least for the winning model (averaged across participants or for one typical participant). In particular, for the scaled evidence model, it would be informative to compare the scaling of the visual and auditory sensory evidence, in order to get an appreciation of the efficiency of the visual and auditory confidence computations.

In the original manuscript, we provided the best fitting parameters for each participant for each model in Supplementary Tables 9 – 10. We note, however, that there is a lot of information in these tables which is not easy to navigate or interpret. In the revised manuscript, we have therefore provided a table that summarises the best-fitting noise and boundary parameters for the free-exponent model across tasks in Table 3. We present boundary parameters, which are derived from the scaling factors, *k* and *m* parameters, in this table rather than raw parameter estimates as it allows for easier comparison of differences across modalities.

Minor comments:

- page 1: it seems that there is a typo in the email address of the corresponding author (missing one 'c').

The corresponding author's email address has been updated in the revised manuscript. We thank Reviewer #3 for picking up this error!

- the manuscript sometimes refers to Locke et al. (2021) when I think they mean 2022 (e.g. lines 139, 175).

We thank Reviewer #3 for noting the error. We missed some references to Locke et al.'s pre-print (2021) which hadn't been updated to the accepted article (2022) in the original manuscript. In the revised manuscript, we have cross-checked all the intext references with the reference list.

- Figure 2B: personally I would have matched the y-range in the bottom panel to that of the top panel (i.e. between 0 and 1) to emphasize that the evidence for category 2 in the different SDs task does not go down to zero.

Figure 2B has been updated to match the y-axis range in the revised manuscript.

- page 17: I understand that the different categories and intensity levels were all interleaved within a block of trials, rather than having a single intensity level (and both categories) in some mini-blocks. Maybe this could be clarified in the methods section.

Reviewer #3's interpretation of the experimental design is correct. To clarify, we have added the following sentence to the Methods in the revised manuscript:

> *Within a testing block, an equal number of test stimuli were sampled from each category and each intensity level (i.e., 15 trials per cell of the design). This meant that within a testing block, participants saw stimuli from both categories presented at every intensity level. The order of both category and intensity level were randomised.* [Lines 368 - 372]

- page 25, line 509: the parameter 'mu_2' does not appear in the equations above. Is this because in Equation 3, 'mu_2' = - 'mu_1'?

That is correct. For the different means task, $\mu_2 = -\mu_1$ and $\sigma_1 = \sigma_2$ so the calculation of the log posterior probability ratio can be simplified as $\frac{2x\mu_1}{\sigma^2+\sigma_1^2}$. We are happy to provide further details about the derivation of this Equation if Reviewer #3 would like.

Also, it would probably be nice here to clearly state the difference between 'sigma' and 'sigma_1'.

In the revised manuscript, we have added text to clarify the difference between σ and σ_1:

> *Where μ_1 and σ_1 are the mean and standard deviation of the category 1 distribution, σ_2 is the standard deviation of the category 2 distribution and σ is the fitted value of measurement noise, approximating the amount of sensory uncertainty in the observers' internal representation of the stimulus (see Equation 2).* [Lines 579 – 582]

- page 28, line 571: the parameter t that refers to the total number of trials here was used before (page 24, line 489) to refer to the sensory modality.

We thank Reviewer #3 for highlighting the double use of *t*. In the revised manuscript, we now use *M* to refer to the sensory modality and *t* to the total number of trials.

- page 29, Equations 9 and 11: there is a missing 'B' on top of the sum sign.

We have added *B* to the top of the sign sum in Equations 10 and 12.

- Figure 5: can the authors match the range of y-axis across all panels, like what they have done for Figure 4?

In the revised manuscript, we have updated Figure 5 to match the range of the y axis across panels.

- Figures 7A and 7B: why not remove the circular symbols, that are redundant with the solid lines, so that the square symbols can be more visible?

We chose to use the circular symbols for the model predictions (in addition to the solid lines) to highlight that we were plotting binned data however, we agree

with Reviewer #3 that this makes the square symbols more difficult to see. We have, therefore, removed the circular symbols in the revised manuscript. We have also added some text to the figure caption to highlight the binning of the model data. We have left the circular symbols in the supplementary figures.

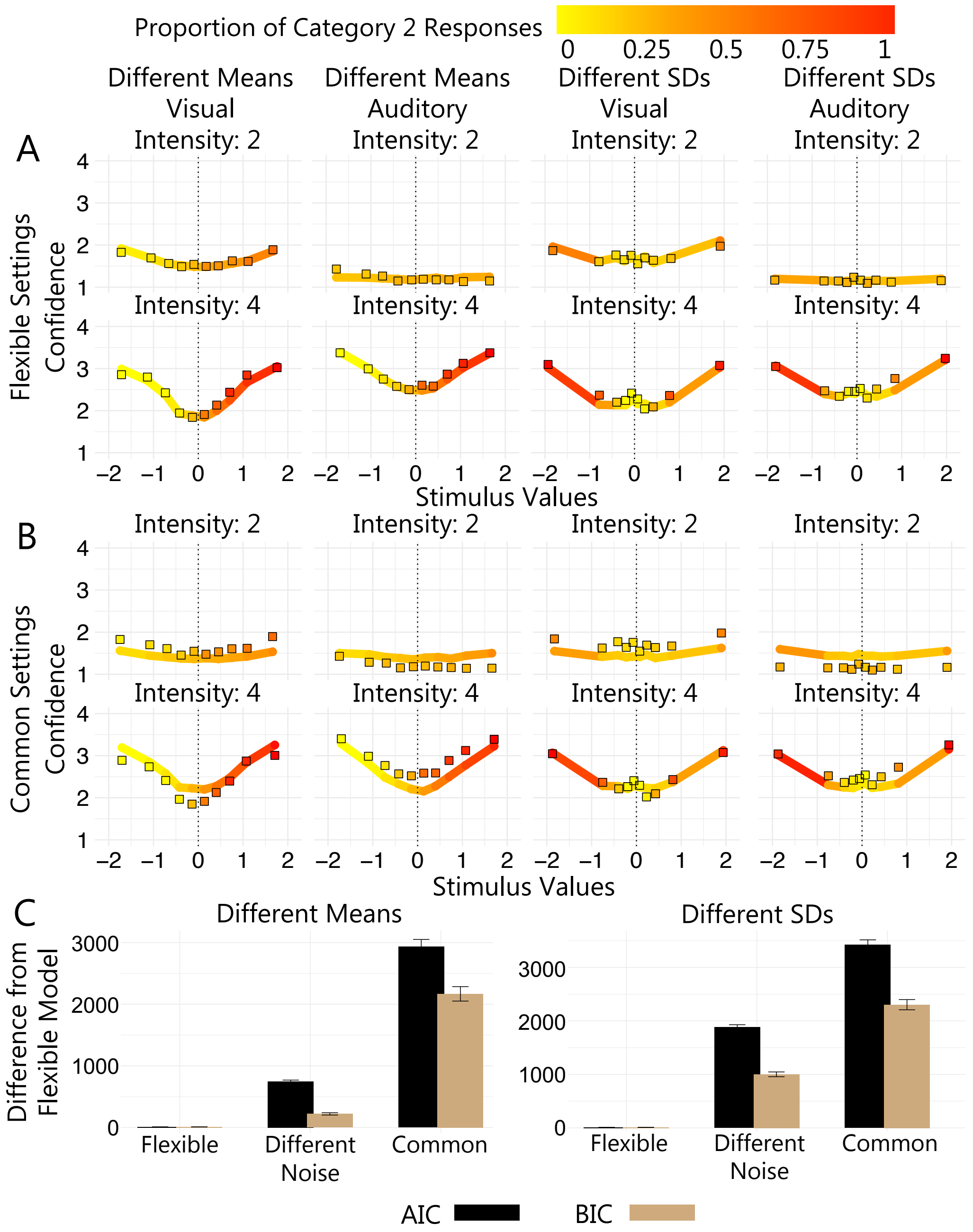

Fig 8. Parameter Settings Across Modalities. *(A) and (B) show mean confidence and proportion of category 2 responses (colour scale) for binned stimulus values. Square plotting symbols show means for experimental data and solid lines show means for (A) flexible settings model predictions and (B) common settings model predictions. Columns show different versions of the task and rows show different intensity levels. For clarity the plots include results for intensity levels 2 and 4 only. Difference in summed AIC (black) and BIC (brown) between the flexible settings model and the other models with different parameter settings in (C) the different means task and (D) different SDs task.*

- Figures 7C and 7D: from the text (page 50, lines 996 and 997), it looks like the labels “offset noise” and “different noise” have been inverted.

> **We thank Reviewer #3 for noting this error. In the revised manuscript, we chose to remove these models to simplify the model recovery analysis and model comparison results. For further clarification, please see the second half of comment #1 from Reviewer #2.**

- Supplementary information, line 247: I think “confidence” is on the y-axis, not the x-axis.

> **We thank Reviewer #3 for their attention to detail! In the revised Supplementary Materials, we have fixed this error:**
>
> > *In the figures below, we do not collapse over category and confidence and instead show confidence on the y axes and use colour to show proportion of Category 2 responses.* [Lines 472 - 474]
